# Functional redundancy and metabolic flexibility of microbial communities in two Mid-Atlantic bays

**DOI:** 10.1093/ismeco/ycag021

**Published:** 2026-02-02

**Authors:** Jojy John, Maximiliano Ortiz, Pierre Ramond, Barbara J Campbell

**Affiliations:** Department of Biological Sciences, Clemson University, Clemson, SC 29634, United States; Department of Biological Sciences, Clemson University, Clemson, SC 29634, United States; Genomics & Bioinformatics Facility, Clemson University, Clemson, SC 29634, United States; Department of Marine Biology and Oceanography, CSIC, Institute of Marine Sciences (ICM), Barcelona, Catalunya 08003, Spain; Department of Biological Sciences, Clemson University, Clemson, SC 29634, United States

**Keywords:** estuarine microbiome, metabolic flexibility, metagenome assembled genomes, metatranscriptomics, microbial functional redundancy

## Abstract

Functional redundancy (FRed) is expected to buffer ecosystems against change, yet it has rarely been characterized in natural systems. How changes in microbial metabolisms, activity, and FRed in ecosystems are influenced by temporal, spatial, and environmental patterns is especially unclear. Here, we analyzed paired metagenomic and metatranscriptomic datasets from surface water samples collected in the Chesapeake and Delaware Bays, USA. These adjacent estuaries experience similar climatic conditions but differ in nutrient availability, salinity, and other environmental factors. We reconstructed 345 high quality metagenome assembled genomes and assessed their metabolic flexibility, and the extent of gene encoded (potential) and expressed (realized) FRed as a function of environmental drivers, microbial lifestyle (free living vs. particle attached), and gene function. The microbiomes exhibited high metabolic flexibility, reflecting their potential, and in many cases, realized gene expression, to exploit diverse energy sources, ranging from organic carbon substrates to trace gases. Potential and expressed FRed varied across seasons, lifestyles, and gene functions, and was structured within each bay by environmental factors such as temperature, salinity, and concentrations of phosphate, silicate, and chlorophyll a. These findings highlight variability in community-level metabolism, and FRed across estuarine microbiomes, shaped by environmental conditions, seasonality, and lifestyle, and provide insights into how these communities may respond to future perturbations.

## Introduction

Microbial communities are central to ecosystems, driving processes such as resource uptake, biomass production, decomposition, and nutrient cycling, which support biodiversity and biogeochemical cycles [1, 2]. Widespread anthropogenic pressures, including heavy metal contamination, agricultural salinization, and industrial pollution, have been shown to disrupt microbial community structure and reduce metabolic activity, thereby altering biogeochemical cycling and ecosystem functioning [3, 4]. These impacts are increasingly evident across terrestrial, freshwater, and marine ecosystems worldwide. However, the mechanistic links between microbial diversity and ecosystem functioning remain poorly understood. The resistance and resilience of an ecosystem to disturbances may partly depend on functional redundancy (FRed), i.e. the number of species that perform similar ecological roles within the ecosystem [5, 6]. FRed may maintain ecosystem stability by providing new taxa with similar functional roles but better fitness under the altered environment [6, 7]. Quantifying FRed may thus be crucial for predicting ecosystem functioning amid species loss and disturbances [6, 8]. The FRed of microbial communities can be quantified at both the genomic and gene expression levels [9, 10].

The ability of microbes to metabolize various compounds are encoded by genes that can be identified in genomes and transcriptomes [11]. These marker genes can be used as “effect traits,” outlining the role of a microbial taxon in ecosystem functioning. Functionally redundant taxa may then differ in their niches, allowing them to perform the same function under contrasting environmental conditions [12]. Linking microbial functional potential to environmental conditions provides a means to connect genomic potential with ecological function [13]. Functional diversity, therefore, reflects the joint influence of effect traits and response traits; the latter determines how taxa respond to environmental change [14]. Newer metrics and frameworks allow estimations of community level diversity and FRed across multiple effect traits [15, 16], and explore FRed patterns across biomes and communities.

While microbial functions in the ocean can remain stable across spatial gradients despite taxonomic turnover [17, 18], they often covary with community composition over time [19], reflecting differing spatial and temporal controls on microbial dynamics. These contrasting patterns have led to the hypothesis that microbial metabolic functions and taxonomic composition in marine environments are shaped by distinct ecological processes [20]. Similarly, the abundance of metabolically distinct microbial groups in coastal ecosystems has been shown to fluctuate throughout the year [21]. Previous research also highlights the influence of spatial, temporal, and phylogenetic scales, as well as ecological mechanisms such as resource availability, dispersal, and competitive exclusion, in shaping functional diversity and redundancy [19, 22]. These findings underscore that microbiomes respond variably to disturbances [23, 24] and suggest that communities in proximity, yet originating from distinct water masses, can harbor vastly different functional potentials [19]. While multi omics approaches now enable the direct estimation of these properties [22, 25], the mechanisms driving their variability across habitats, such as estuaries, remain unclear.

Estuaries are dynamic ecosystems where changes in environmental factors can cause community disturbances [26]. They may be ideal for studying environmental patterns that influence microbial FRed. The Chesapeake and Delaware Bays are adjacent estuaries that share the same climatic conditions and tidal patterns [27] but are ecologically distinct. With its simple structure (funnel shaped) and high tidal amplitude, Delaware Bay has a well-mixed water column, characterized by river discharges that peak in spring and reach a minimum in summer [28–30]. Despite high nutrient concentrations, primary production is typically limited due to high turbidity and reduced light penetration [31]. The Chesapeake Bay has multiple riverine sources but a deeper central channel, which results in partial stratification, increased light penetration, uneven salinity distribution, and a varying chemical state, including hypoxia and acidification in the summer [32]. Seasonal changes in abiotic and biotic conditions have previously been reported in both bays, which may alter FRed [33–35].

Using twenty (ten from each bay) metagenomes and two metatranscriptomes per metagenome (20 from each bay) with a genome resolved approach, we quantified microbial composition, metabolic flexibility, and FRed from Chesapeake and Delaware Bay in relation to environmental variation. We hypothesized that differences in environmental and seasonal conditions, such as salinity gradients and temperature variability, would influence FRed and community-level metabolic flexibility in estuarine microbiomes. Samples were collected from spring and summer across a salinity gradient to capture environmental diversity, thereby maximizing differences between samples [22, 36]. Our study contributes to the assessment of metabolic flexibility and FRed in shaping the response of microbiomes to environmental variability in estuarine ecosystems.

## Materials and methods

### Sample collection, metadata, sequencing, and metagenome assembled genome recovery

Sample collection and associated metadata are further detailed elsewhere [36]. Water samples from Chesapeake Bay (2014) and Delaware Bay (2015) were collected during spring and summer cruises from surface waters (~1.5 m below the sea surface) along longitudinal transects spanning the upper to lower estuary and sequentially filtered through 0.8 μm and 0.22 μm pore size filters to obtain two distinct size fractions: particle-associated (PA, retained on the 0.8 μm filter) and free living (FL, everything less than 0.8 μm and greater than 0.22 μm). Metagenomic and metatranscriptomic library construction and sequencing, along with the workflow for data assembly and preprocessing, are previously described [35, 36]. For an overview of samples and details of assembly and metagenome assembled genome (MAG) (with >70% completeness and < 5% contamination) recovery, refer to the supplementary information and Tables S1 and S2.

### Functional annotation, metabolic prediction, and gene expression

Gene predictions and annotations from MAGs were made using Prodigal [37], followed by Metabolic V.4.0 [11] and Distilled and Refined Annotation of Metabolism, [38]. Different classes of CAZymes and their associated modules involved in carbohydrate utilization were identified using dbCAN3 [39].

MAGs were also searched against a custom set of 51 metabolic markers for specific metabolic processes ([40, 41]; Table S3), predicted using DIAMOND v.0.9.22 [42] with default settings (query coverage >80%). The markers covered the major pathways for aerobic and anaerobic respiration, energy conservation from both organic and inorganic compounds, carbon fixation, nitrogen fixation, and phototrophy [40, 41, 43, 44]. To minimize bias from incomplete genomes, the copy number of key metabolic genes in each MAG was converted to a percentage of its total genes and normalized against the mean genome completeness of MAGs from the same order using R [43]. For more details on MAG level metabolic flexibility please see the supplementary information.

To assess expression, we analyzed the metatranscriptomic patterns of metabolic markers and CAZymes from the current study as described previously [22]. For detailed information, please see the supplementary information.

### Microbial functional redundancy

Integrating trait based and taxonomic abundance data has helped reveal mechanisms of species coexistence [45, 46] and predict ecological stability and ecosystem buffering [47, 48]. We employed an approach based on species annotation, incorporating functional traits and species abundance, to quantify FRed [16, 25, 45, 48]. Focusing on traits from different metabolic processes (supplementary information), we created a genome trait table using the Hidden Markov Model hit number from METABOLIC V4.0 [11], which included ≤187 genes, and generated a distance matrix of MAG traits using Euclidean distance. Based on the abundance of MAGs across samples and their trait-based distance, we computed FRed using the function *uniqueness ()* from the R package “Adiv” [15, 16]. FRed usually ranges from 0 to 1; when FRed is 0, all the species in a community are considered functionally distinct, and when it is 1, all the species are functionally identical, i.e. they harbor the same trait(s) [15, 16, 49]. To estimate expressed FRed, we mapped the gene variants predicted from Metabolic V4.0 [11] for each MAG with the metatranscriptomes of each sample, thus obtaining a trait table of the MAGs present in each sample. Read mapping was performed using Bowtie 2 [50]. The data were normalized to transcripts per million (TPM) value and used as an analogous representation for the expressed traits in each MAG. Supplementary Table S4 (sheet 3) lists the categories and genes used.

In addition, we assessed the presence of specific energy traits and substrate utilization traits among MAGs to estimate potential and realized FRed across environmental gradients. Here, we defined energy traits to encompass genes directly involved in cellular energy conservation, including those involved in electron transfer, redox reactions, and ATP generation. In contrast, substrate utilization traits comprised genes associated with the metabolism of diverse organic carbon substrates. FRed was also quantified for CAZymes and for different metabolic modules, including carbon monoxide (CO), hydrogen (H_2_), nitrogen (N), and sulfur (S) transformations.

### Statistical analysis

We evaluated the statistical relationships between FRed and environmental factors using linear regression models in R [51]. First, we evaluated the normality of the data using Q-Q plots generated with *qqnorm ()* and *qqline ()* in R [51]. We then used a linear regression model from *lm ()* in R [54] to statistically assess the relationship between FRed and environmental factors [52], including size fraction, bay, season, salinity, and other abiotic factors. Highly correlated environmental variables were identified by analyzing the univariate association between FRed and all available metadata (https://www.bco-dmo.org/dataset/565451) using a linear model and were removed prior to analysis. Based on these results, a refined multivariate model was then predicted using the subset of variables, which included season, salinity, and temperature. This model was then validated by plotting the fitted values against the observed values. Quantified potential and expressed FRed across different seasons, bays, and energy vs. substrate utilizations, different metabolic modules and CAZymes were statistically tested for significant differences using a Wilcoxon Rank Sum Test [53].

## Results

### Abundance and diversity of metagenome assembled genomes

**Figure 1:**
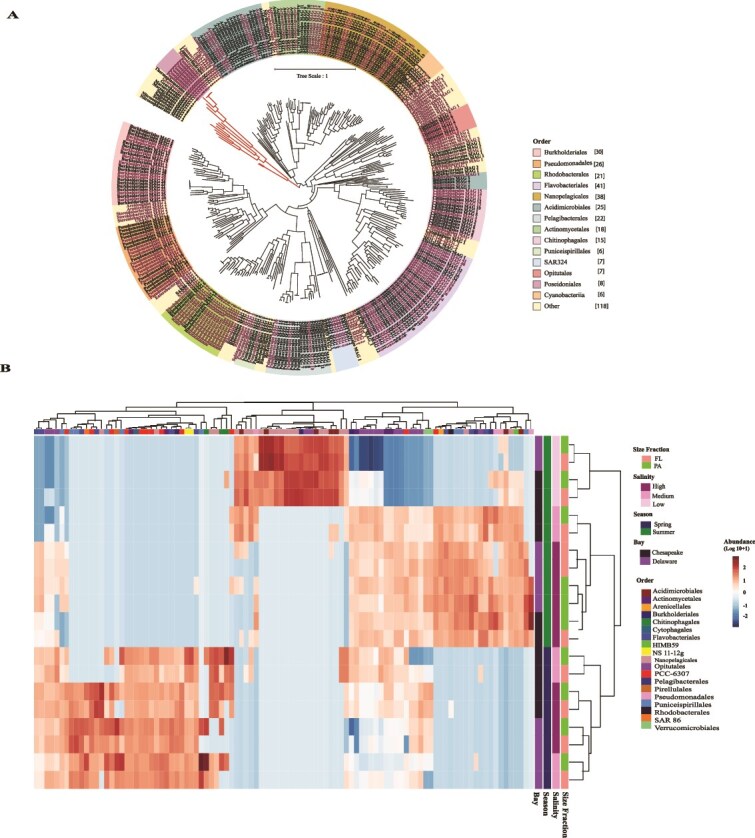
Bacterial and archaeal MAGs from the Chesapeake and Delaware bays. (A) Phylogenetic tree of MAGs built by GTDB-tk based on 120 conserved single copy marker genes for bacteria and 122 for archaea. The background color of each clade indicates the order to which each MAG belongs, and the number in parentheses in the legend indicates the number of MAGs per indicated order. The branches indicate bacteria (black) and archaea (red) from the current study. (B) Normalized abundance of top 100 MAGs in the indicated metagenomes estimated through CoverM.

After dereplication to species level (ANI > 95%), 345 MAGs (179 MAGs originated from Delaware and 168 from the Chesapeake Bay) recovered with >70% completeness and <5% contamination. Nineteen were classified as Archaea; the remainder were classified as Bacteria (Fig. 1A). The read coverage of the MAGs across the metagenomes/contigs ranged from 45% to 70% of total reads (Table S2). The taxonomic composition, as inferred from MAGs, significantly varied across seasons and salinities (PERMANOVA; *R*^2^ = 0.30, F = 10.64, *P* = .001; Figs. 1B and S1). Overall, low salinity samples were dominated by *Burkholderiales*, while *Pelagibacterales* dominated medium and high salinity samples. PA MAGs had significantly larger genomes than FL MAGs (mean genome size: PA = 2.35 Mb; FL = 1.28 Mb; Wilcoxon rank-sum test, *P* < .001; Cliff’s δ = 0.77). Specific details are found in supplementary information.

### Functional strategies

#### Community-level metabolic flexibility

**Figure 2:**
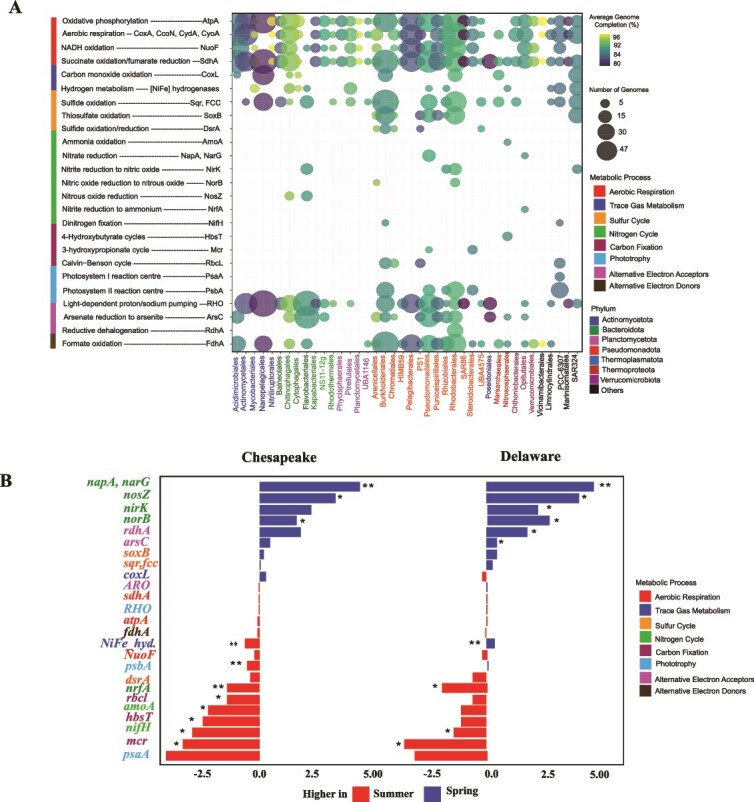
Community-level metabolic potential of microbial communities from the Delaware and Chesapeake bays. (A) Distribution of key metabolic genes across microbial orders in the Delaware and Chesapeake bays. Bubble size represents normalized gene abundance, with values normalized by the average genome completeness of genomes assigned to each order. (B) Log₂ fold changes of metabolic marker genes across all MAGs present in spring vs. summer. Bars represent the difference in mean log₁₀ abundance of all MAGs encoding each gene. Positive log₂ fold change indicates higher abundance in spring, whereas negative log₂ fold change indicates higher abundance in summer. Significance of differences between seasons was assessed with Wilcoxon tests (^*^P < .05; ^**^P < .01). Data are shown separately for each bay.

Profiling of 51 metabolic marker genes in recovered MAGs highlighted their functional capabilities, with contrasting strategies encoded across *Actinomycetales, Burkholderiales, Flavobacteriales,* and *Rhodobacterales* (Figs. 2A and S2). Significant effects of bay, season, and their interaction on metabolic marker gene abundance were observed (Fig. S3). S metabolism genes (*dsrA, fcc, sqr, soxB*) were broadly distributed, with *dsr* more abundant in summer, irrespective of bays (Fig. 2B). N cycling genes exhibited seasonal partitioning: *nrfA* was detected only in Delaware Bay at medium to high salinity levels in summer, *nifH* appeared in both bays during summer, while *nosZ, norB, napA*, and *narG* were restricted to the spring. Genes for rhodopsins and H_2_ metabolism (NiFe hydrogenases) were prevalent across bays and seasons, with aerobic H₂ uptake and formate oxidation capacity observed in multiple orders. CO oxidation (*coxL*) was enriched in PA fractions and increased with salinity, particularly in Chesapeake Bay. MAGs contained key genes for several other core metabolisms (Table S4, Sheet2). See supplementary information for more details.

The expression of these genes varied by season, salinity, and bay (Fig. S4). Most of the variation was explained by season, with salinity contributing moderately and bay having the smallest effect (Fig. S5). Variance partitioning confirmed that season accounted for the largest proportion of gene expression variability, followed by salinity and bay. Seasonal differences were evident within each bay, with several genes (e.g. *nifH*, *psbA*, *coxL*) enriched in summer relative to spring. Salinity further structured gene distributions, with high salinity samples clustering together, while medium and low salinity samples showed more variable profiles (Fig. S4).

#### Carbohydrate active enzymes

**Figure 3:**
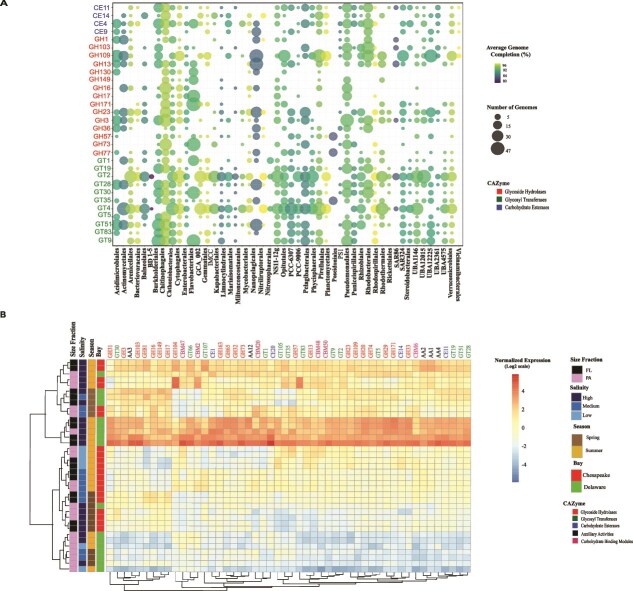
CAZymes in the Chesapeake and Delaware Bay MAGs. (A) Most abundant CAZymes indicated at the order level. CAZymes were defined into different enzyme families, including GH (glycoside hydrolase), GT (glycosyl transferase), and CE (carbohydrate esterase), which are shown on the y axis. Bubble size represents the normalized gene abundance, and hits were normalized based on the genome completeness of MAGs from each order. (B) Top 50 most highly expressed genes from different CAZy families. Rows (row scaled) represent samples, and columns represent individual CAZyme genes, grouped and color coded by family: GH (glycoside hydrolases), GT (glycosyl transferases), CE (carbohydrate esterases), AA (auxiliary activities), and CBM (carbohydrate binding modules). Colors within the heatmap indicate normalized expression values (log₂ scale). Sample annotations (e.g. salinity, season) are also shown above the columns. Sample annotations above the columns denote size fraction (FL; PA), salinity (high, medium, low), season (spring, summer), and bay (CP = Chesapeake Bay; DE = Delaware Bay), which are differentiated by distinct colors as shown in the legend. Different CAZyme families are also color coded according to their functional classes.

The presence of CAZymes genes varied by salinity, regardless of season and bay, with higher numbers in PA fractions (Figs. 3A, S6, and  S7). The dominant CAZymes irrespective of salinity included glycoside hydrolases (GH) families 2, 73, 23, 107; glycosyl transferases (GT) modules 51, 4, 28, 2; carbohydrate esterases (CE) 4; carbohydrate binding modules (CBM) 50, 44; and auxiliary activity (AA) classes 4, 3, and 2 (Figs. S6 and S7). Statistical analysis of the seasonal and spatial variation in CAZymes abundance revealed that season and salinity are key drivers shaping the distribution of carbohydrate active enzymes across both bays. Significant salinity effects were found during the summer for several CAZymes families, including two from CE (11, 14), seven from GH (1, 103, 109, 13, 36, 73), and four from GT (28, 30, 4, 83, 9) (*P < .05*; Fig. S8). This was further supported by expression analysis, which revealed that multiple CAZyme genes exhibited the strongest differences across seasons, followed by salinity and bay (Fig. 3B). Specifically, GH transcripts were more abundant in Chesapeake Bay, particularly during the spring and under low salinity conditions. In contrast, Delaware Bay was enriched in GTs, AAs, and CBMs transcripts, especially in summer and under medium to high salinity conditions.

### Microbial functional redundancy

**Figure 4:**
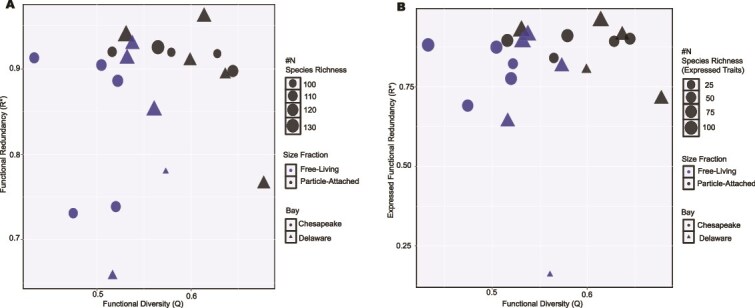
FRed vs. functional diversity. (A) Potential FRed vs. functional diversity. (B) Expressed FRed vs. functional diversity. Red and black colors represent PA (G08) and free-living (L08) communities, respectively. Circles and triangles distinguish the bays as Chesapeake and Delaware, respectively, and the size of the shapes represents species richness. The y axis scale of both panels is not identical due to differences in the values.

**Figure 5:**
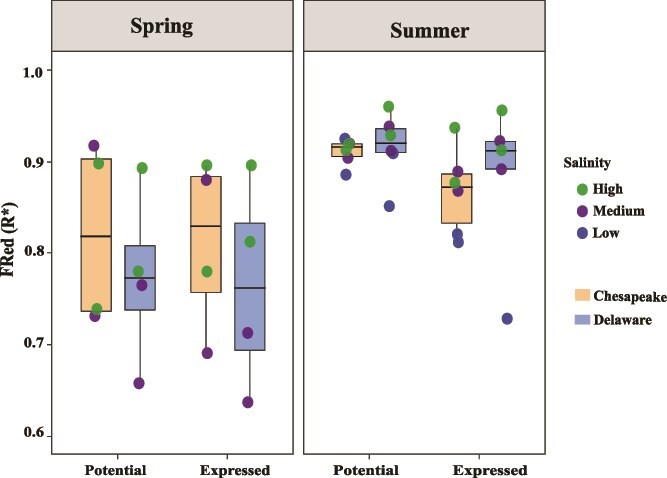
Potential and expressed FRed in the Chesapeake and Delaware bays across seasons and salinity regimes. Comparison of potential and expressed FRed between seasons and bays. FRed data points are colored according to salinity regime in each season. Statistical comparisons across salinity levels were limited due to small sample sizes. In both panels, the box colors represent the two bays as indicated in the legend. One low salinity Delaware Bay sample from the expressed FRed dataset was excluded from the figure due to a low value (FRed = 0.08).

Microbial FRed, assessed using a metabolic gene trait table comprising up to 187 genes, showed a weak and non-significant correlation with functional diversity (Q; Pearson’s r = 0.13, *R*^2^ = 0.02, *P =* .58) but was moderately and significantly positively correlated with species richness (N; Pearson’s r = 0.47, *R*^2^ = 0.22, *P = .037*; Fig. 4). The Chesapeake microbiome exhibited a slightly higher, though non-significant, FRed (0.88 ± 0.07) compared to the Delaware microbiome (0.85 ± 0.09). Potential FRed (0.87 ± 0.08) was generally higher than expressed FRed (0.80 ± 0.17). Overall, FRed (both potential and expressed, unless specified) was structured by season, salinity, and size fraction (Figs. 4 and 5). FRed was higher in summer than in spring, with a significant difference in potential FRed between seasons in both bays (*P* < .002; Fig. 5). In spring, FRed was higher in the Chesapeake than in the Delaware microbiome, whereas the opposite pattern was observed in summer (Fig. 5). FRed also differed between size fractions, with the PA community showing significantly higher values (*P* < .04) than the FL community (Fig. 4). Salinity further influenced both potential and expressed FRed across bays and seasons (Fig. 5). Samples from higher salinity sites generally exhibited greater FRed values, particularly during summer. Although statistical comparisons among salinity groups were limited by small sample sizes, a PERMANOVA model including salinity, bay, and season explained 50% of the total variation in functional trait structure (F = 3.77, *P* = .004), and linear regression modeling also identified salinity as a significant positive predictor of FRed (*P* = .037 for Pot FRed and *P* < .007 for expressed FRed, respectively).

Potential FRed exhibited a strong positive correlation with temperature for FL and PA communities (Table 1). Additional positive correlations were observed with salinity, number of cells, phosphate, and silicate, while nitrate and chlorophyll showed negative, but non-significant relationships in both size fractions (Fig. S9). Expressed FRed showed a significant correlation with salinity in the PA fraction and a negative correlation with nitrate in the FL fraction (Table 1). Linear regression models indicated that significant predictors of potential FRed were temperature (*P* < .007), size fraction (*P* < .001), season (*P* < .001), and salinity (*P* < .037). The overall model also explained 76% of the variability in the data (Adjusted *R*^2^ = 0.7609, F = 8.523, *P* < .0001286), suggesting that the change in FRed is primarily structured by the environmental parameters listed above. However, linear regression models on expressed FRed revealed that salinity (*P* < .007) and ammonium (*P* < .03) were significant predictors (Adjusted *R*^2^ = 0.3877, F = 5.01, *P <* .01226), accounting for an overall 38.8% variability in the data (Table S5).

**Table 1:** Correlations of FRed with environmental variables in PA and FL microbial fractions. Potential FRed represents the predicted FRed from metagenomic potential, while Expressed FRed represents FRed measured from metatranscriptomic expression.

FRed	Factor	PA		FL	
		*R* ^2^	*P*-value	*R* ^2^	*P*-value
Potential FRed	Temperature	0.651	.041	0.912	.0001
Expressed FRed	Salinity Nitrate	0.654 0.408	.04 .04	0.36 −0.949	.06 .005

#### Functional redundancy of specific metabolic traits

**Figure 6:**
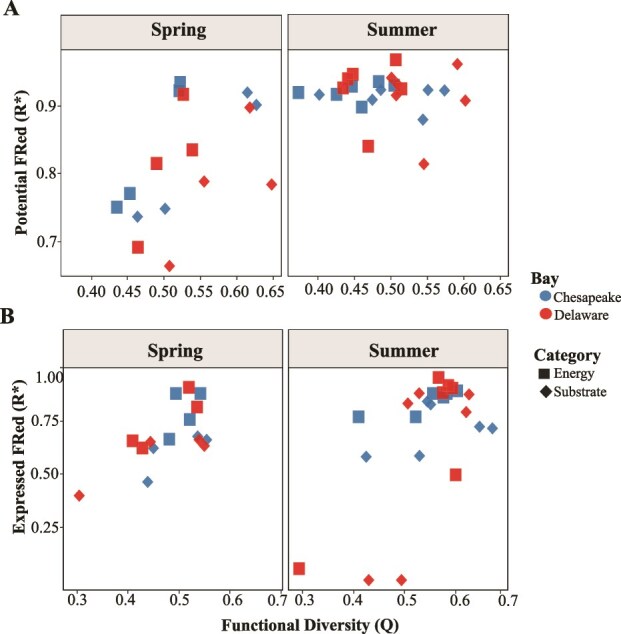
FRed of energy vs substrate utilization. (A) Potential FRed of energy metabolism and substrate utilization genes across the Chesapeake and Delaware bays during spring and summer. (B) Expressed FRed of energy metabolism and substrate utilization genes across the Chesapeake and Delaware bays during spring and summer. Squares represent energy metabolism genes, and diamonds represent substrate utilization genes in both plots. The analysis includes 37 genes for energy metabolism and 47 genes for substrate utilization. The y axis scale of both panels is not identical due to differences in the values.

We determined the seasonal differences in FRed between energy and substrate utilization of the MAGs between the bays separated by salinity (Fig. 6). We defined energy traits as genes directly involved in cellular energy conservation, including those involved in electron transfer, redox reactions, and ATP generation. In contrast, substrate utilization traits comprised genes associated with the metabolism of diverse organic carbon substrates. The distribution of FRed in energy metabolism and substrate utilization traits varied between the bays but was not significant overall. However, season exerted a significant effect on potential FRed in Delaware Bay, with both energy and substrate traits showing higher variability between spring and summer (*P =* .019 and *P* = .010, respectively). In contrast, expressed FRed differed significantly between energy and substrate traits in Chesapeake Bay, with energy traits showing higher redundancy than substrate traits in both spring (*P* = .043) and summer (*P =* .025). Functional diversity (Q) also differed between metabolic categories, with Substrate traits showing significantly higher Q values than Energy traits across all samples (*P =* .0008). This pattern was consistent in both Chesapeake (*P =* .041) and Delaware Bays (*P =* .005), was strongest during summer (*P =* .0047), and showed only a marginal trend in spring (*P =* .059).

We next analyzed potential and expressed FRed among the genes in four different metabolic modules: CO, H_2_, N, and S. Each module represents the complete set of known traits involved in cycling these elements (Table S4, Sheet 3). Potential FRed increased from spring to summer (Fig. S10). Low and medium salinity samples from Delaware Bay displayed a lower potential FRed in all tested modules than high salinity samples. Potential FRed did not differ much between salinities in the Chesapeake Bay, except within the N module, where it increased with salinity. Potential FRed was highest in H_2_ metabolism in both bays in both seasons, followed by CO metabolism in the spring and N metabolism in the summer. Expressed FRed was lower than potential FRed in all instances (Fig. S10A and B). Expressed FRed was higher in CO and S metabolic traits in the Chesapeake than in Delaware Bay and generally increased with salinity (Fig. S10C and D). However, we could not accurately calculate expressed FRed in H_2_ or N metabolic modules due to the low number of transcripts, and statistical analyses were limited by small sample sizes.

**Figure 7:**
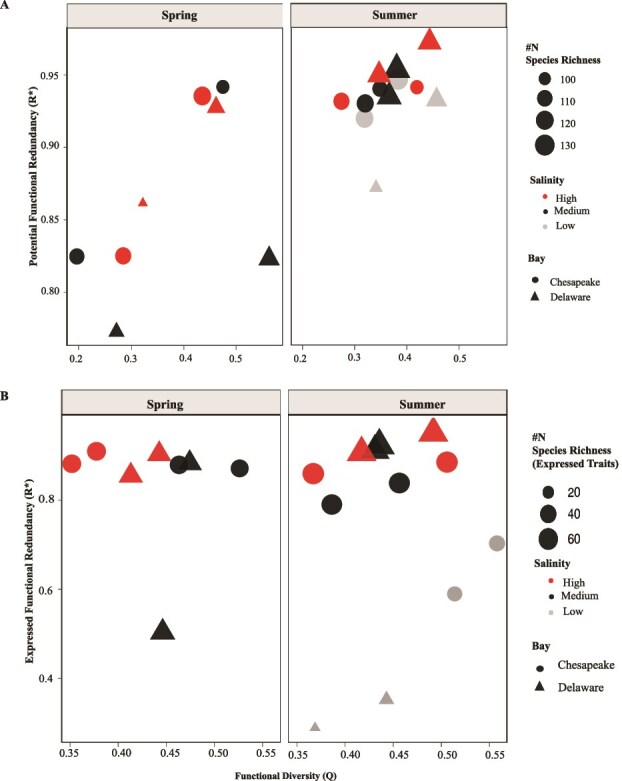
Potential and expressed FRed vs. functional diversity of CAZymes. (A) Potential FRed of CAZymes across all samples from both bays. (B) Expressed FRed of CAZymes from both bays. Circles represent Chesapeake Bay samples, and triangles represent Delaware Bay samples, while colors indicate salinity and symbol size represents species richness. This analysis includes 1548 CAZYmes belonging to different enzyme families, including GH (glycoside hydrolase), GT (glycosyl transferase), and CE (carbohydrate esterase).

Potential FRed derived from CAZyme traits varied significantly between estuaries, seasons, and size fractions (Fig. 7A). FRed increased in summer relative to spring (ANOVA, F = 31.61, *P =* .0003*; Kruskal Wallis, χ^2^* = 6.10*, P* = .0136). Delaware Bay CAZymes exhibited significantly higher overall FRed than Chesapeake Bay (*β* = 0.09 ± 0.04*, P* = .046), primarily driven by a significant summer increase (*P* = .025), whereas seasonal differences in Chesapeake Bay were not significant (*P* = .46). Multiple linear regression analysis (*R*^2^ = 0.88, adjusted *R*^2^ = 0.75*, P =* .004) identified season (*P* = .0003), chlorophyll a (*P* = .022), and size fraction (*P* = .0033) as significant predictors of potential FRed. In contrast, expressed FRed showed similar trends across seasons, bays, and salinity, although none were statistically significant (Fig. 7B). Among all environmental variables, only nitrate concentration emerged as a significant predictor (*β* = −2.6 × 10^−3^, *P* = .0097) for expressed FRed. Functional diversity (Q) showed clear seasonal and salinity patterns. Spring samples were highly variable, while summer samples displayed more uniform functional diversity. Low salinity sites generally had lower Q values, whereas several high salinity Delaware samples showed the highest potential Q. In contrast, expressed Q values were tightly clustered, and none of the tested relationships with Q were statistically significant.

## Discussion

Despite increasing efforts to link microbial genomic potential with expressed traits and community composition, a comprehensive framework integrating these dimensions, together with species richness and environmental drivers, remains underdeveloped [25]. This limits our ability to explore the relationship between microbial metabolisms, potential and realized functional diversity, and FRed across changing habitats. Therefore, understanding how microbial communities respond to environmental variability remains a central challenge in microbial ecology. We demonstrate that community-level metabolic flexibility and FRed vary across seasonal and salinity gradients in estuaries, revealing how environmental variability shapes both the potential and realized functional capacity of microbial communities.

### Use of multiple energy sources

Most of the recovered MAGs from these estuaries were predicted to be primarily heterotrophic organisms capable of metabolizing a diverse range of organic substrates, emphasizing both individual and community metabolic flexibility. The degradation of algal debris, necromass, and host glycans likely plays a crucial role in meeting the energy demands of these microbiomes, particularly in eutrophic estuaries, where stratification, oxygen depletion, and fluctuating organic matter inputs can create temporal and spatial energy limitations [54, 55]. The most abundant and actively expressed CAZyme families detected in our estuarine microbiomes were composed of peptidoglycan lyases (GH23,73, &107) and chitinases (CBM 50 &44), which are primarily involved in the breakdown of necromass, such as cellulose, chitin, and peptidoglycan [54, 55]. This was further supported by the diversity of bacterial orders harboring substrate specific genes for chitin degradation and peptidoglycan turnover. Other families abundant in these estuarine microbiomes target algal polysaccharides, such as fucans and xylan [56] and many orders also encode genes specific to lignin, polyphenol, and host glycan degradation [55, 56]. Together, these findings suggest that heterotrophy and the degradation of complex organic matter are likely primary energy acquisition strategies in these estuarine microbiomes.

In addition to heterotrophic metabolism, many MAGs possessed genes enabling alternative energy acquisition pathways such as photo- and litho- heterotrophy [22, 56, 57]. This metabolic flexibility was reflected in the expressed profiles, which included transcription of genes involved in H_2_ and S oxidation/reduction (*dsrA, fcc, sqr*, and *soxB*). Bacterial taxa from multiple orders *Burkholderiales, Chitinophagales, Flavobacteriales, Pseudomonadales,* and *Rhodobacterales* encoded genes for trace gas and S oxidation/reduction and were detected in both estuaries, consistent with previous observations in marine [41] and terrestrial ecosystems [58]. Chesapeake Bay is historically characterized by algal blooms and increased H_2_ production during late spring and summer [59, 60], while seasonal stratification results in hypoxia and H_2_ release [61]. In Delaware Bay, H_2_ accumulation from algal blooms and urban inputs similarly results in summer hypoxia [62]. The elevated abundance of S oxidation and reduction genes in both bays during summer suggests that microbes can use sulfide as an inorganic or supplemental energy source, as supported by previous studies [63, 64]. Overall, in addition to light driven energy gains [35], the capacity for S, H_2_, and CO oxidation likely provides these primarily heterotrophic microbes with the metabolic flexibility to persist under conditions of organic substrate limitation.

### Patterns of functional redundancy

Multiple studies across diverse ecosystems, including plant, animal, and marine, have demonstrated the relationship between species diversity and FRed [18–20, 65–67]. However, many of these studies focused on single environmental contexts, leaving the effects of multiple ecological conditions largely unexplored [8, 16]. Direct comparisons are also limited by the lack of consistent approaches for FRed estimation across ecosystems. Here, we found multiple estuarine environmental variables influence FRed, including season. During spring, lower and more variable FRed coincided with higher functional diversity, indicating that a wide range of metabolic niches was available and occupied by functionally dissimilar taxa. In contrast, summer conditions, characterized by elevated temperature, variable stratification, hypoxia, and increased nutrient inputs [61, 68], likely restricted the number of available metabolic niches. Under such conditions, microbial communities likely converged toward shared functional repertoires, resulting in higher potential and expressed FRed. Oxygen depletion and nutrient enrichment during summer [61, 69, 70] may have reduced the diversity of usable electron donors and acceptors in both bays, forcing microbial taxa to rely on a smaller set of core metabolic pathways and thereby increasing redundancy [61, 71–73]. Consistent with this pattern, we observed greater variability in potential FRed during spring than in summer across CO, H₂, N, and S metabolic modules. The observed decline in expressed relative to potential FRed during summer further suggests that microbes maintain viability by downregulating nonessential processes while preserving core energy metabolism [74, 75].

We also observed that FRed was generally highest at marine salinities, where nutrient concentrations and available organic substrates were lowest. Under these conditions, resource limitation and osmotic stress likely constrained the diversity of metabolic niches, leading microbial taxa to rely on a narrower suite of core energy yielding pathways and thus increasing FRed. Consistent with this pattern, the composition and abundance of CAZyme groups decreased with increasing salinity during summer, likely reflecting the predominance of labile carbohydrates in low salinity environments and more recalcitrant carbohydrates at higher salinities [76]. Consistent with our findings, salinity strongly influenced microbial composition and function in the Mediterranean Sea [19] and drives transcriptional shifts in these estuaries, where *Rhodobacterales* vary in photoheterotrophic, carbon, N, and S metabolism and anaerobic, S reducing pathways in Chesapeake Bay waters [22, 77]. Higher variability was observed in potential FRed during spring than in summer in both bays across different salinity regimes. In Delaware Bay, this may reflect the co-occurrence of autochthonous estuarine bacteria and freshwater taxa introduced by river inflows. During spring, strong freshwater pulses combined with the bay’s small size, shallow depth, and tidal mixing cause rapid salinity shifts [27]. The increased freshwater input also transports freshwater bacteria into mid salinity regions, where some taxa remain metabolically active but express more stress related genes [22]. Similarly, in Chesapeake Bay, high variability in potential FRed during spring may be linked to the seasonal succession of bacterial communities driven by rapid temperature increases, nutrient pulses, and freshwater inflow, resulting in major restructuring of microbial assemblages [34, 78]. As conditions stabilize toward summer, only a subset of phylogenetic groups likely persist; those able to tolerate low oxygen and stratified environments [78], leading to higher FRed in medium and high salinities observed in this study. Previous studies also reported clear seasonal differences in dominant bacterial groups and variations in microbial diversity and abundance along salinity gradients in both bays [33, 34, 78, 79]. Altogether, these findings suggest that environmental stress and community turnover during spring drive the observed variability in potential FRed across salinity regimes.

Furthermore, FRed was consistently higher in energy utilization traits than in substrate utilization traits, reflecting microbial strategies to sustain energy generation under nutrient limited conditions and to recover from environmental disturbances [80, 81]. Supporting this, our analyses of metabolic genes and transcripts revealed that individual MAGs possess and actively express flexible metabolic repertoires, enabling them to utilize multiple energy sources. In contrast, lower FRed in substrate utilization traits indicates metabolic specialization and resource partitioning under nutrient limited conditions [82]. This pattern aligns with our CAZyme-derived FRed results, where variability in available carbon sources increased Q and reduced overall FRed. In Delaware Bay, the high heterogeneity of organic matter [76] likely promoted the coexistence of taxa specialized for different substrate types, thereby reducing redundancy in substrate related functions. The negative correlation between phosphate concentration and expressed FRed further supports that nutrient limitation suppresses nonessential metabolic activity [83, 84]. Together, these results suggest that while energy metabolism is broadly redundant and resilient, substrate degradation is more specialized and environmentally constrained.

The FRed of CAZymes exhibited pronounced spatial and seasonal structure, with potential FRed consistently higher in summer than in spring across all salinity regimes, while spring exhibited greater variability among salinity groups. Expressed FRed remained high across medium and high salinity samples in both seasons but declined markedly at low salinity during summer. This likely reflects the influence of organic matter heterogeneity and bloom derived inputs, as frequent mixed phytoplankton blooms in Delaware Bay increase the diversity and lability of available polysaccharides [69, 76]. Such conditions can favor metabolic specialization, allowing taxa to exploit distinct carbohydrate niches while maintaining overall functional overlap in CAZyme profiles [85]. This interpretation is consistent with the pronounced spring variability in functional diversity (Q) across salinity in Delaware Bay and the more uniform Q values observed in summer in both bays, alongside the dominance of CAZyme families involved in degrading diverse detrital organic matter, including bloom derived, plant, and eukaryote derived substrates such as chitin, cellulose, and peptidoglycan [54, 55]. In contrast, expressed CAZyme FRed remained consistently high across both bays and seasons, consistent with previous reports that show specific bacterial groups drive the post bloom degradation of phytoplankton derived organic matter in marine and estuarine systems [86, 87]. This pattern may result from the sustained presence and recycling of algal derived polysaccharides and low molecular weight sugars that persist into summer, supporting continuous transcription of carbohydrate active enzymes despite shifts in nutrient and oxygen conditions [85, 88]. Several studies further support the notion that byproducts of spring phytoplankton blooms persist into summer, providing a long-term source of labile organic substrates that sustain microbial enzymatic activity [55, 89, 90]. In addition, summer blooms are often distinct from spring blooms, typically dominated by Cyanobacteria or mixed phytoplankton assemblages that further contribute to the pool of organic matter available for microbial degradation [89]. Together, these findings suggest that CAZyme FRed is primarily governed by the availability, diversity, and lability of organic matter, rather than by broad seasonal, salinity, or other abiotic and biotic factors.

Finally, microbial lifestyle introduced an additional dimension to FRed patterns. PA taxa are key degraders of complex organic matter, whereas FL microbes primarily process dissolved substrates [34]. The significantly larger genomes in PA communities compared to FL ones are consistent with the greater functional potential required for complex substrate degradation [91]. Nutrient conditions, such as phosphate and silicate availability, may further influence these lifestyle patterns by selecting for phylogenetic groups adapted to specific nutrient regimes [22, 77]. For instance, higher phosphate and silicate concentrations may favor taxa with larger genomes and more versatile metabolisms, contributing to the positive correlation between these nutrients and potential FRed. Collectively, these results highlight that both FRed and metabolic flexibility underpin the adaptive capacity of estuarine microbiomes, enabling them to maintain ecosystem processes in the face of fluctuating salinity, nutrient availability, and other environmental stressors.

## Conclusion and outlook

Our findings suggest that estuarine microbial communities adapt through community-level metabolic flexibility and FRed, enabling them to withstand or recover from environmental disturbances. Specifically, these communities meet their metabolic needs through photoheterotrophy and lithoheterotrophy [41, 92], and exhibit high FRed, allowing similar ecosystem functions to be maintained despite seasonal changes in metabolic gene composition, possibly through the mixing of ecotypes or niche differentiation. They also show enhanced FRed in energy utilization genes, traits essential in nutrient or substrate limited environments. With ongoing environmental changes and the expansion of anoxic zones [93], these traits may help sustain key ecological processes. However, low redundancy communities, such as those in spring, which exhibit higher functional diversity but lower FRed, may be more vulnerable to functional or taxonomic losses. Importantly, higher FRed does not always equate to greater ecosystem resilience [25], highlighting the need for in-depth studies on the relationships between ecosystem stability, process efficiency, and multifunctionality in relation to FRed.

## Supplementary Material

John_etal_26_Supplementary_information_ycag021

Suppl_Table_S4_updated_ycag021

## Data Availability

The metagenomes, metatranscriptomes, and MAGs are available in NCBI under the umbrella project PRJNA432171. All the detailed codes and calculations are available on our GitHub page: https://github.com/Campbelllab-bioinfo under FRed-and-metabolic-flexibility-of-estuarine-microbiome.
