# Supplementary material for: Functional redundancy and metabolic flexibility of microbial communities in two Mid-Atlantic bays
Source: ISME Commun. 2026 Feb 2;6(1):ycag021. doi: 10.1093/ismeco/ycag021 (PMC12911934; doi:10.1093/ismeco/ycag021)
Supplement: John_etal_26_Supplementary_information_ycag021 [file john_etal_26_supplementary_information_ycag021.pdf]

Supplementary Information

Functional redundancy and metabolic flexibility of microbial communities in two Mid Atlantic bays

Jojy John[1], Maximiliano Ortiz[1,2], Pierre Ramond[3] and Barbara J Campbell[1*]

[1] Department of Biological Sciences, Clemson University, Clemson, SC, USA

[2] Clemson University Genomics & Bioinformatics Facility, Clemson University, Clemson, SC, USA

[3] Institute of Marine Sciences (ICM), Department of Marine Biology and Oceanography, CSIC, Barcelona, Catalunya, 08003, Spain

*Corresponding author

Supplementary Materials and Methods

Sample collection and metadata

Surface (~1.5 mbsf) water samples were collected using a rosette sampler during three cruises in 2014 and two in 2015 of longitudinal transects of the Delaware and Chesapeake Bays, respectively, as detailed in Ahmed et al. [1]. Briefly, collected water samples were sequentially filtered through 0.8 and 0.22 µm pore size filters to obtain two distinct size fractions. The metadata and the sampling locations can be found at (**https://www.bco-dmo.org/dataset/565451**) and Ahmed et al. [1].

Metagenome and metatranscriptome sequencing

Metagenomic and metatranscriptomic library construction and sequencing were previously described [1,2]. In this study, a total of 20 (ten from each bay) metagenomes and 2 metatranscriptome per metagenome (20 from each bay) were used from the original samples as described in Ahmed et al. [1]. The Chesapeake samples originated from low (0.01-0.06 PSU), medium (15-20 PSU), and high (27-31 PSU) salinities during the spring and summer of 2015 while the Delaware samples were from low (0.019-0.03 PSU), medium (15-22 PSU), and high (29-30 PSU) salinities during spring and summer of 2014 [1,2]. Metagenomes from low salinity samples from the Spring were excluded from this study because of their low coverage values (<4%) (Supplementary Table S2).

Metagenomic reads were classified with Kaiju V 1.10.0, and bacterial and archaeal reads were used to determine relative abundances at the order level with default settings using the _nr database from Kaiju's index databases [3]. For each data set, binning was done with MetaWRAP and refined into MAGs using MetaWRAP: Bin_refinement_module [3,4]. De-replication was performed with dRep v3.1.1 [5] and completeness and contamination were assessed with CheckM V1.0.7 [4]. The abundances of each MAGs in the metagenome were estimated using CoverM [6]. MAG taxonomic classification was performed with the Genome Taxonomic Database (GTDB Tk V2.1.1, [7]).

Metagenome binning, dereplication and analysis

Metagenomic binning was performed separately for each assembly. Metagenome-assembled genomes (MAGs) were constructed using MetaWRAP v1.1 [3]. Default parameters were used for all steps unless otherwise explicitly mentioned. The contigs were binned with MaxBin2 V2.2.5, MetaBAT2 V2.12.1, and CONCOT v1.1.0 and the refined into MAGs using MetaWRAP: Bin_refinement_module [3] with > 70% completeness and < 5% contamination and these MAGs were retained for further analysis. For quality assessment and dereplication, we used CheckM V1.1.10 (lineage_wf default parameters, [4]) and dRep (v3.4.0) based on 95% average nucleotide identity (ANI), respectively [5]. The normalized (CPM) abundances of each de replicated MAG were calculated based on the percentage of MAG reads over Kaiju defined bacterial and archaeal reads in each Chesapeake Bay and Delaware Bay metagenome using CoverM 'genome' with the

following settings: --min-read-percent-identity 0.95 --min-read-aligned-percent 0.75 --trim-min 0.10 --trim-max 0.90 [6].

Gene expression analysis

Metagenome assembled genomes (MAGs) were mapped to metatranscriptomes, and feature count tables were generated and consolidated according to previously identified metagenomic gene clusters [8]. For downstream analysis, only metatranscriptomes with ≥10,000 reads mapped to genes within a MAG were included. Variance stabilizing transformation of count data was performed using DESeq2 [9]. Principal component analysis (PCA) of the transformed counts (plot PCA, DESeq2) was used to visualize sample clustering by Season, Bay, and Salinity. Gene level annotations were used to subset metabolic marker genes and CAZYme genes, and log_2 transformed normalized counts were visualized as heatmaps. For large gene sets, the most variable genes were selected to improve the interpretability of the heatmaps [9,10].

Metatranscriptome mapping and analysis

A functional gene catalog of all MAGs was prepared to calculate expressed FRed. We retrieved all genes/sequences identified as traits using Metabolic V 4.0 [11] and metatranscriptomic reads were mapped to these functional traits for each MAG using Bowtie2 [8]. The data was normalized to transcripts per million (TPM) value and used as an analogous representation for the expressed traits in each MAG.

MAG- level metabolic flexibility

Metabolic potential at the MAG level was assessed using curated marker genes representing major energy acquisition strategies, including heterotrophy, lithotrophy, phototrophy, and autotrophy. Heterotrophic potential was inferred from the presence of core respiratory genes (e.g., *AtpA, NuoF, CoxA, SdhA*). Lithotrophic potential was defined by the presence of genes involved in sulfur (e.g., *Sqr, SoxB, DsrA*), nitrogen (e.g., *NarG, NapA, NirK, NosZ*), hydrogen, carbon monoxide, and trace gas oxidation. Phototrophic potential was inferred from the presence of photosystem genes (*PsaA, PsbA*) or rhodopsins (*RHO*). Autotrophic potential was inferred from the presence of carbon fixation markers (e.g., *RbcL, HbsT*). MAGs were classified into lifestyle categories based on the combination of marker genes detected within each genome as photo-lithoheterotroph, photoheterotroph, lithoheterotroph ,heterotroph and putative autotroph.

Supplementary Results

Microbial composition in bays

A collection of diverse microbial communities reflective of different orders, featuring *Pelagibacterales, Burkholderiales, Flavobacteriales, Rhodobacterales, Acidimicrobiales,*

Rhizobiales, Micrococcales, and *Nanopelagicales* as dominant groups were found in these two bays using read based analysis. The microbial community composition in Chesapeake Bay (CP) and Delaware Bay (DE) varied with salinity levels. In summer low salinity samples, *Burkholderiales* was the dominant order in both particle attached (PA, >0.8 micron, G08) and the free living (FL, <0.8 micron, L08) fractions in both bays. Medium and high salinity conditions showed a more balanced distribution among *Pelagibacterales, Flavobacteriales, and Rhodobacterales* in both bays regardless of the size fraction. During spring, *Rhodobacterales, Flavobacteriales, and Pelagibacterales* were dominant across the medium and high salinity in both size fractions of both bays. High salinities contained increased *Synechococcales* in Chesapeake compared to Delaware and a consistent dominance of *Pelagibacterales* in both bays. The abundance of many orders increased with increasing salinities in the bays, including the *Synechococcales* in the Chesapeake and *Sporadotrichia* in the Delaware.

Abundance and diversity of MAGs

In summer, *Burkholderiales* MAGs dominated in both size fractions at low salinity in both bays, followed by *Acidimicrobiales* and *Nanopelagicales*. Medium salinity samples were dominated by *Pelagibacterales* and *Acidimicrobiales*, while high salinity samples featured *Pelagibacterales, Flavobacteriales,* and *Rhodobacterales* (Figure 1). In spring, *Pelagibacterales, Rhodobacterales*, and *Pseudomonadales* were prevalent at medium and high salinities. Overall, *Nanopelagicales, Pelagibacterales, Acidimicrobiales,* and *Flavobacteriales* were key players in summer, while *Pelagibacterales, Rhodobacterales*, and *Pseudomonadales* dominated at medium and high salinity in both bays during spring (Figure 1B). The binning recovered zero MAGs from several orders, which were identified through read based taxonomy, including *Alteromonadales, Bacillales Cellvibrionales, Clostridiales, Corynebacteriales, Micrococcales, Oceanospirillales* and *Streptomycetales.*

There was a positive correlation between many environmental factors and MAG abundance (Figure S1). RDA analysis revealed that 85% of the variance in the MAG abundances in the bays was structured by different explanatory variables, including salinity, temperature, and phosphate concentrations (all with p values <0.001), chlorophyll a, silicate, and nitrate concentrations and size fraction (all with p values <0.05).

Functional strategies

Community and MAG level metabolic flexibility

The profiling of 51 marker genes in the recovered MAGs gave insight into their functional capabilities. Many MAGs, including representatives from *Actinomycetales, Burkholderiales*, *Flavobacteriales,* and *Rhodobacterales*, encoded multiple strategies, indicating metabolic flexibility at the genome level (Figure 2A, Table S4, Sheet 1). Results from ART ANOVA revealed

significant effects of bay, season, and the interactive effects of both on the abundance of microbial metabolic marker genes (Figure S2).

At the MAG level, metabolic potential was distributed across multiple lifestyle categories, with photo-lithoheterotrophs (n = 122) representing the largest group, followed by photoheterotrophs (n = 75), lithoheterotrophs (n = 46). Multiple metabolic strategies frequently co-occurred within individual genomes, as many MAGs classified as heterotrophic based on core respiratory markers also encoded genes involved in sulfur, nitrogen, hydrogen, and trace gas oxidation, as well as phototrophic processes (Figure S2).

The major gene contributors for sulfur metabolism (*dsrA*, *fcc*, *sqr,* and *soxB*) were found in five orders irrespective of bay (Figure 2A). The number of MAGs carrying *dsr* (sulfide reduction/oxidation) was higher during the summer compared to the spring. Nitrogen cycle genes were more prevalent in PA than the FL fraction. Genes responsible for nitrate reduction to ammonia (*nrfA*) were only seen in Delaware Bay under medium and high salinity samples during summer, while dinitrogen fixation (*nifH*) was seen during summer in both bays. In contrast, nitrous oxide reduction (*nosZ*), nitric oxide reduction to nitrous oxide (*norB*) and nitrate reduction (*napA* and *narG*) were seen only during spring in both bays. The ammonia oxidation gene (*amoA*) was detected only in the order *Marsarchaeales*, indicating archaeal involvement in nitrogen metabolism. (Figure 2). Genes involved in nitrogen metabolism and formate oxidation were more prevalent during spring and summer, respectively. MAGs within six orders encoded genes for microbial rhodopsins in both bays, regardless of season and bay (Figure 2B).

Genes involved in hydrogen metabolism were higher in the PA than the FL fractions of both bays in both seasons, where 25 MAGs possessed *NiFe hydrogenase* genes, which were 23% and 31% of the MAGs in the Chesapeake and Delaware Bays, respectively (Figure 2B). Several subclasses of NiFe dehydrogenases were observed in the community, particularly groups 1d, 1l, and 2a, all responsible for aerobic H_2 uptake, as well as group d, responsible for formate oxidation to H_2. These genes were encoded by MAGs within five orders from both bays (Figure 2). Similarly, CO oxidation genes were abundant in PA fractions and increased with salinity (*coxL,* 29% and 6% in the CP and DE Bays, respectively) and encoded in MAGs within four orders (Figure 2B). The MAGs also contained key genes in several other core metabolisms (Table S4, sheet 2).

Carbohydrate active enzymes and substrate utilization

Members of *Acidimicrobiales* and *Nanopelagicales* were the most dominant contributors in low salinities. *Nanopelagicales, Flavobacteriales* and *Cytophagales* were the prominent contributors in medium salinities, and *SAR324* and *Rhodobacterales* were in high salinities (Figure S7. MAG families prominent in the Delaware Bay were somewhat different from those in the Chesapeake Bay. *Nanopelagicales* and *Burkholderiales* possessed a high number of CAZYmes under low salinity, and *Vicinamibacterales* and *Burkholderiales*, in medium salinities and under high salinity (Figure S7).

Substrate specific gene profiling showed that the major CAZYme encoded functions involved the degradation of xylans, chitin, starch, and xyloglucan, with chitin degrading genes being the most abundant (Figure S11). Taxonomically, *Chitinophagales*, *Flavobacteriales*, and *Pseudomonadales* exhibited the highest number of specific substrate genes, while *Rhodospirillales*, *Puniceispirillales*, and *Pelagibacterales* had the fewest (Figure 3,S7). GH genes found in our MAGs were associated with the degradation of disaccharides, complex polysaccharides, and polyphenols. MAGS containing carbohydrate esterase and polysaccharide lyase genes utilized fewer substrates than MAGs with other substrate associated genes, mainly those encoding proteins involved in polysaccharide use.

Supplementary Discussion

Microbial composition of the surface waters

A read based analysis of the analyzed metagenomes found 30 orders of non-rare bacteria, while our MAG based approach revealed 50 bacterial orders and five archaeal orders. This discrepancy is common in metagenome analysis [12]; less taxonomic information can be retrieved from short sequence reads, leading to a superficial view of the microbiome. In turn, genome resolved analysis can result in a comprehensive view of the dominant members of microbial communities [12].

The microbial communities from the Chesapeake and Delaware bays are structured differently, underscoring both habitat specific characteristics and seasonal variation. Specifically, environmental parameters, including salinity, temperature, and size fraction [2,13], shape microbial composition. In the present study, microbes from the order *Burkholderiales* dominated the lower salinity regions of both bays, consistent with previous reports [13,14]. Similarly, *Pelagibacterales*, *Rhodobacterales*, *Rhodospirillales*, *Flavobacteriales*, and *Acidimicrobiales* were abundant in both size fractions from medium and high salinity regions of both bays, aligning with earlier findings [13-16]. Among other taxonomic groups, *Pelagibacterales* were dominant in medium and high salinities but contained distinct genomospecies, suggesting the bays support different ecotypes adapted to different salinity gradients[14,17].

Supplementary Table S1. Summary of samples used in the current study. The last two rows are list of sample names of the current study, here, CPBay= Chesapeake Bay; DEBay = Delaware Bay; Spr = Spring; Sum = Summer; the numbers following bay name and season indicate salinity in PSU; G08 = >0.8 µm, and L08 = <0.8 µm size fraction respectively.

Bay	Chesapeake	Delaware
Seasons	Spring and Summer	Spring and Summer
Salinity	Low (0.1-15 PSU)<br>Medium (>15-22 PSU)<br>High (>22 PSU)	Low (0.1-15 PSU)<br>Medium (>15-22 PSU)<br>High (>22 PSU)
Spring	CPBay_Spr15G08<br>CPBay_Spr15L08<br>CPBay_Spr31G08<br>CPBay_Spr31L08	DEBay_Spr20G08<br>DEBay_Spr20L08<br>DEBay_Spr30G08<br>DEBay_Spr30L08
Summer	CPBay_Sum06G08<br>CPBay_Sum06L08<br>CPBay_Sum15G08<br>CPBay_Sum15L08<br>CPBay_Sum27G08<br>CPBay_Sum27L08	DEBay_Sum019G08<br>DEBay_Sum019L08<br>DEBay_Sum22G08<br>DEBay_Sum22L08<br>DEBay_Sum29G08<br>DEBay_Sum29L08

Supplementary Table S2. Overview of metagenome read coverage of all MAGs in the indicated sample used in the current study. Metagenomes from low salinity samples from the Spring were excluded from this study because of their low coverage values (<4%). Here, total reads, representing the total number of sequencing reads generated from a metagenome, mapped reads represent the subset of total reads that were aligned to a reference contig/MAGs, and coverage is a measurement of how well the contigs are represented by the sequenced reads.

Sample	Mapped Reads	Total Reads	Coverage
CP_Spr15G08	5497474	11118414	49.44%
CP_Spr15L08	38215011	54011070	70.75%
CP_Spr31G08	12798267	31158252	41.08%
CP_Spr31L08	20416715	36277314	56.28%
DE_Spr20G08	33964433	64466868	52.69%
DE_Spr20L08	6282464	15047522	41.75%
DE_Spr30G08	7276955	16380548	44.42%
DE_Spr30L08	8681175	21664408	40.07%
DE_Sum019G08	13759962	55870026	24.63%
DE_Sum019L08	7537227	22518206	33.47%
DE_Sum22G08	10902604	26669760	40.88%
DE_Sum22L08	16222487	26054748	62.26%
DE_Sum29G08	7053884	21507640	32.80%
DE_Sum29L08	7720265	16733718	46.14%
CP_Sum06G08	30212492	102894128	29.36%
CP_Sum06L08	29810014	70508712	42.28%
CP_Sum15G08	4726292	12279432	38.49%
CP_Sum15L08	11757097	20480666	57.41%
CP_Sum27G08	3640507	13123130	27.74%
CP_Sum27L08	7699248	19826342	38.83%

Supplementary Table S3. List of the fifty one core metabolic marker genes used in the current study and their associated cellular process

Sl. Number	Cellular Process	Genes
1	Aerobic respiration	*coxA, ccoN, cyoA, cydA, atpA, SdhA, and nuoF*
2	Trace gas oxidation	NiFe hydrogenase, coxL, FeFe hydrogenase, *MmoA*, *PmoA,cyc2, and IsoA*
3	Sulfur cycle	*fcc, sqr, dsrA, sor, soxB,* asrA
4	Nitrogen cycling	*amoA, nifH, narG, napA, nirS, nirK, nrfA, nosZ, nxrA, norB, hzsA*
5	Carbon fixation	*mcr, rbcl, aclB, acsB, hbsT, hbsC*
6	Phototrophy	*psaA, psbA, microbial rhodopsins(4)*
7	Alternative electron acceptors	*arsC, rdhA,omcB, mtrB, ygfK,* aro
8	Alternative electron donor	*FdhA, cooS, frdA*

Supplementary Table S4. Distribution of traits observed in the indicated MAGs and their associated metabolic genes and cycles.

Please find the excel file in supplementary data.

Supplementary Table S5. Linear regression model using the lm() function in R estimating the relationship between FRed and environmental factors. **A.** summarizes the regression coefficients, standard errors, t-values, p-values, and significance levels for the factors which are significant. Significant codes correspond to 0 ‘’ ***0.001*** ‘’ *0.01* ‘’ 0.05 ‘.’ 0.1 ‘ ’ 1 and **B**. is the model fit statistics.

A.

**Term**	**Estimate**	**Std. Error**	**t- value**	***P***
(Intercept)	0.4619	0.2457	1.88	0.0797
Size Fraction	0.0483	0.0105	4.61	0.0003***
Season_Spring	-0.1445988	0.0366063	-3.950	0.000732 ***
Temperature	0.0119	0.0038	3.147	0.00665**
Salinity	0.0019728	0.0008853	2.228	0.036912*

B.

**Metric**	**Value**
Residual Standard Error	0.03816
Degrees of Freedom	15
Multiple R-squared	0.8621
Adjusted R-squared	0.7609
F-statistic	8.523
F-statistic Degrees of Freedom	11 and 15
p-value	0.0001286

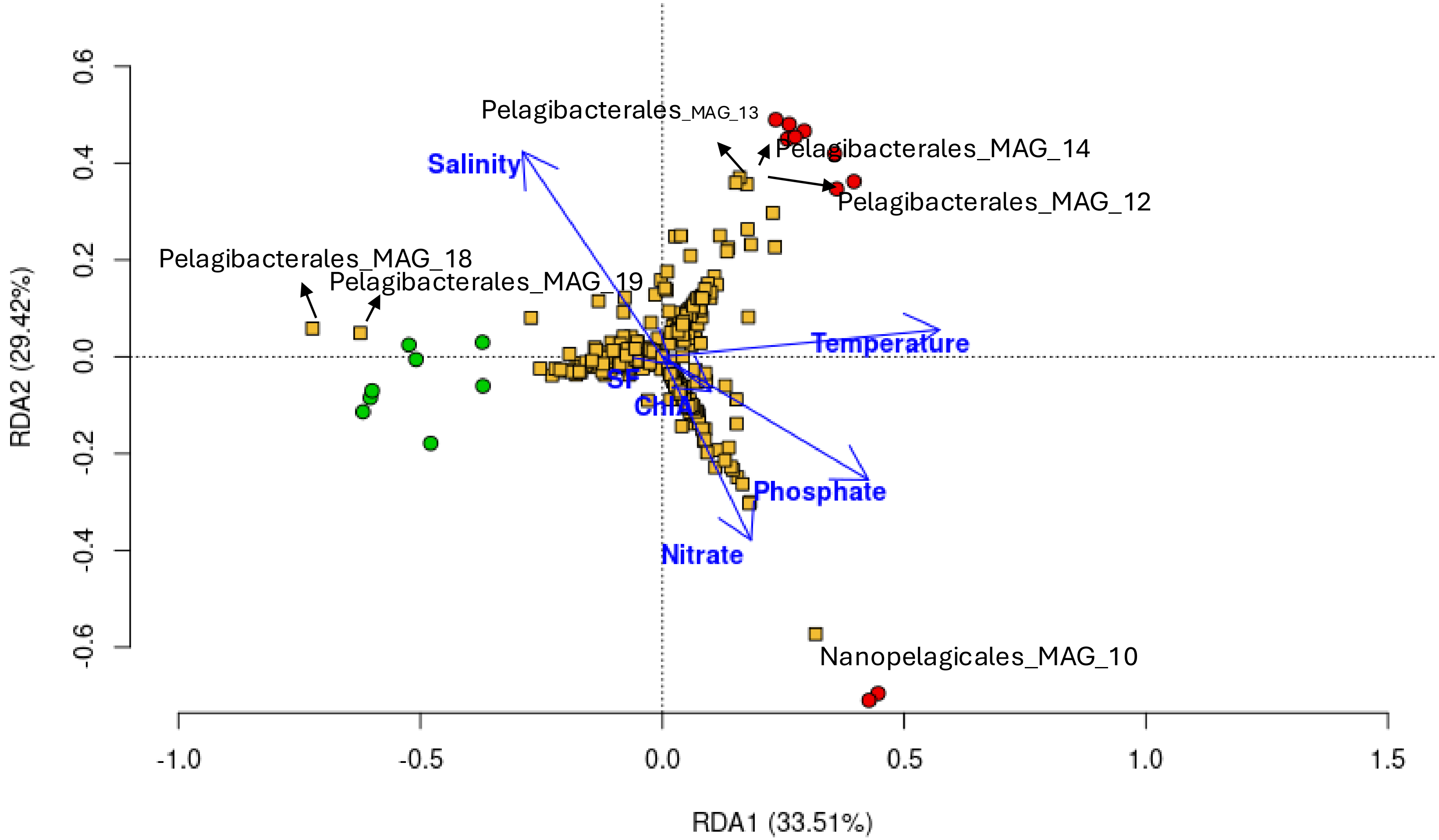

Supplementary Figure S1. Redundancy analysis plot (RDA) of the relationship between the environmental factors (observed metadata) and abundances of recovered MAGs. Vectors are temperature, salinity, nCells, bacterial production, phosphate and nitrate. The yellow rectangles in the center of the plots indicate the MAGs, and the green and red dots indicate spring and summer metagenome samples, respectively. The black arrows and text indicate the taxonomy of the MAG, as indicated. CP = Chesapeake Bay, DE = Delaware Bay, Spr and Sum stand for the season, followed by salinity (06 PSU to 30 PSU) and size fraction (L08 less than 0.8 microns, G08 greater than 0.8 microns).

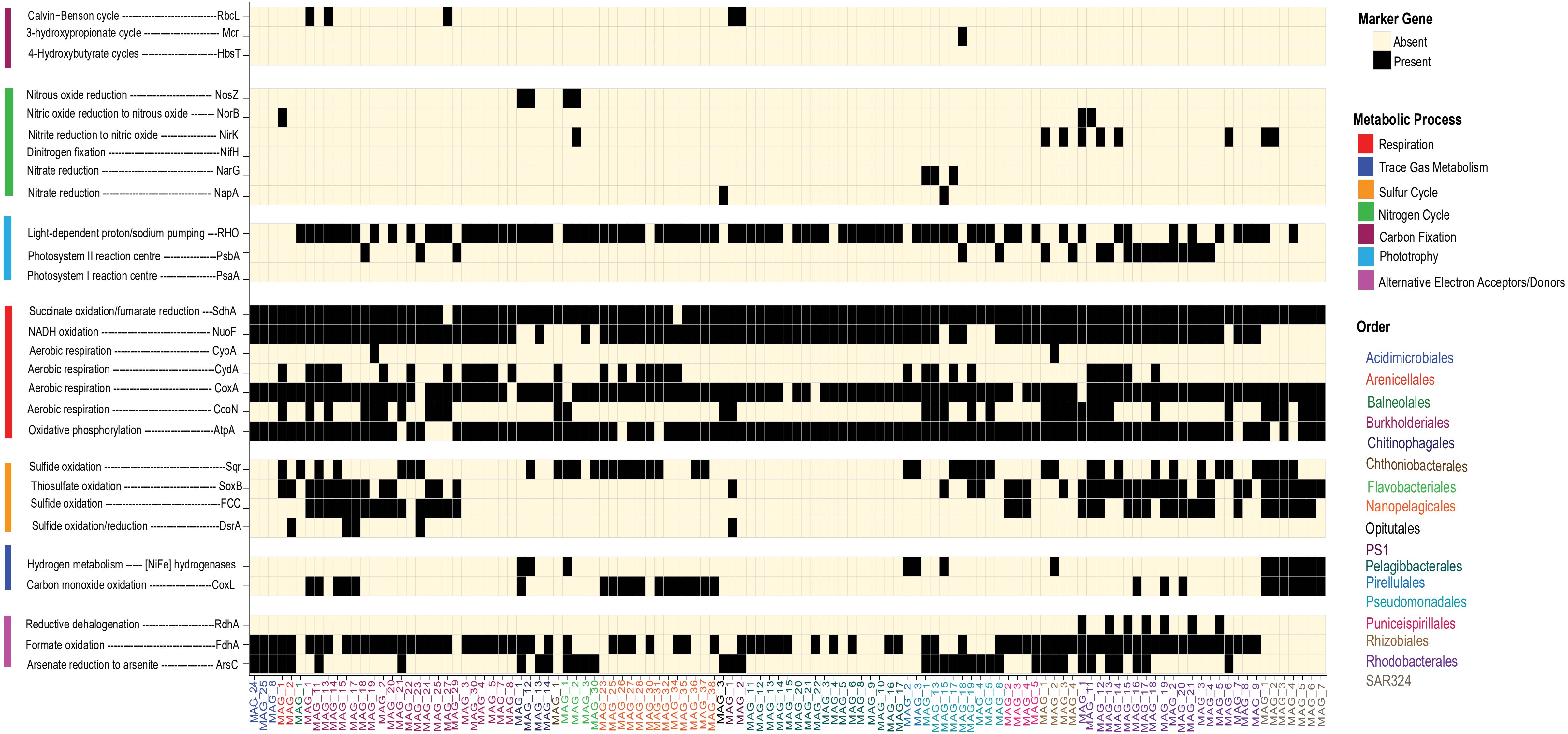

Supplementary Figure S2. Co-occurrence of metabolic marker genes within individual metagenome-assembled genomes (MAGs). Heatmap showing the presence (black) or absence (light) of curated metabolic marker genes across the top 100 MAGs, selected based on marker-gene richness. Columns represent individual MAGs (grouped by taxonomic order), and rows represent metabolic marker genes associated with different metabolic process which is shown in the legend.

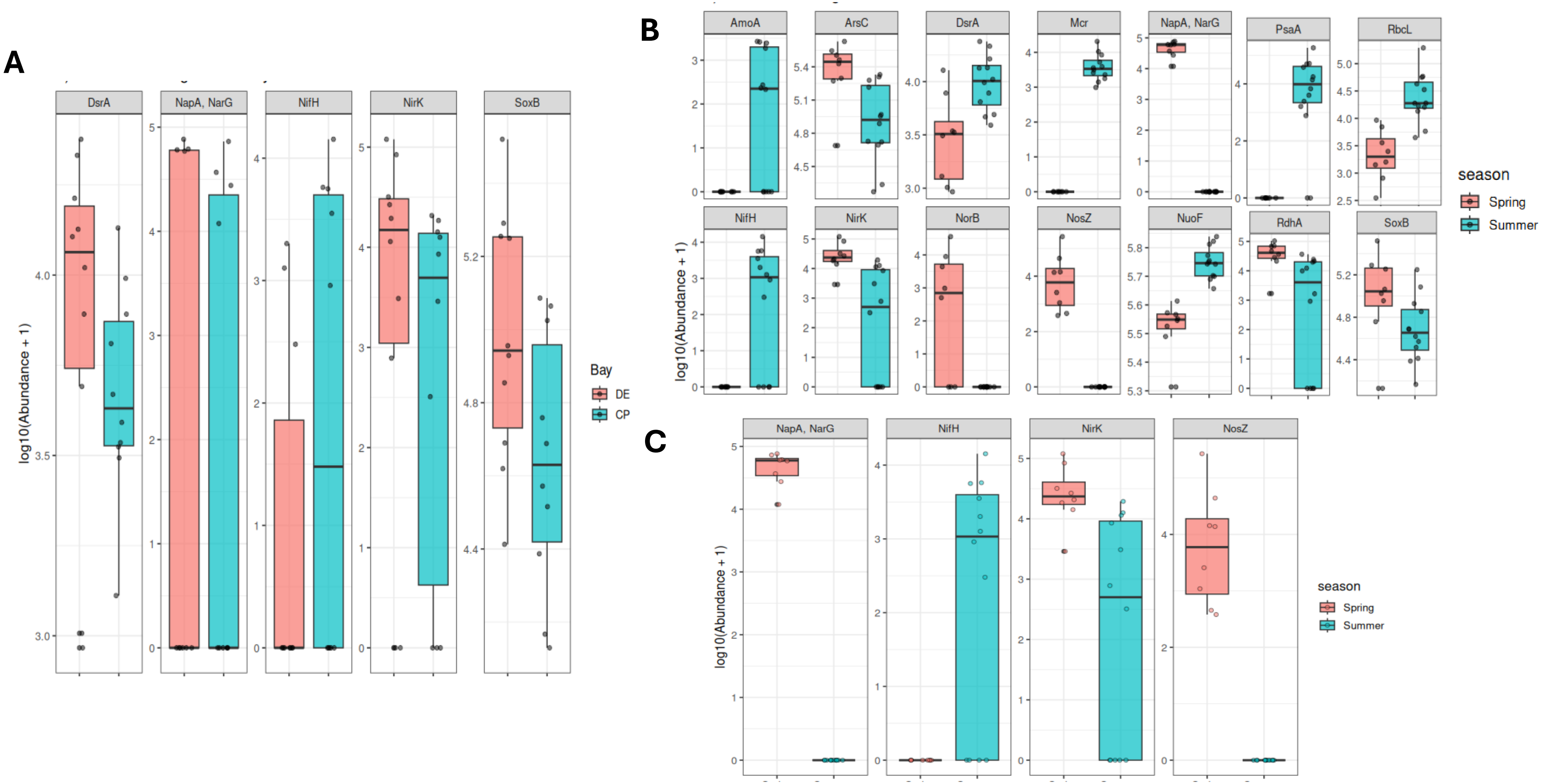

Supplementary Figure S3. Effects of Bay, Season, and their interaction on microbial metabolic marker abundance using ARTS ANOVA. A. Metabolic markers with a statistically significant *Bay* effect, indicating differences in abundance between Chesapeake and Delaware Bays. **B.** Metabolic markers with a statistically significant *Season* effect, highlighting changes in abundance between spring and summer. **C.** Metabolic markers with a statistically significant *Bay* × *Season* interaction, suggesting that seasonal effects differ between the two Bays. Significance was determined based on adjusted p-values (FDR < 0.05)

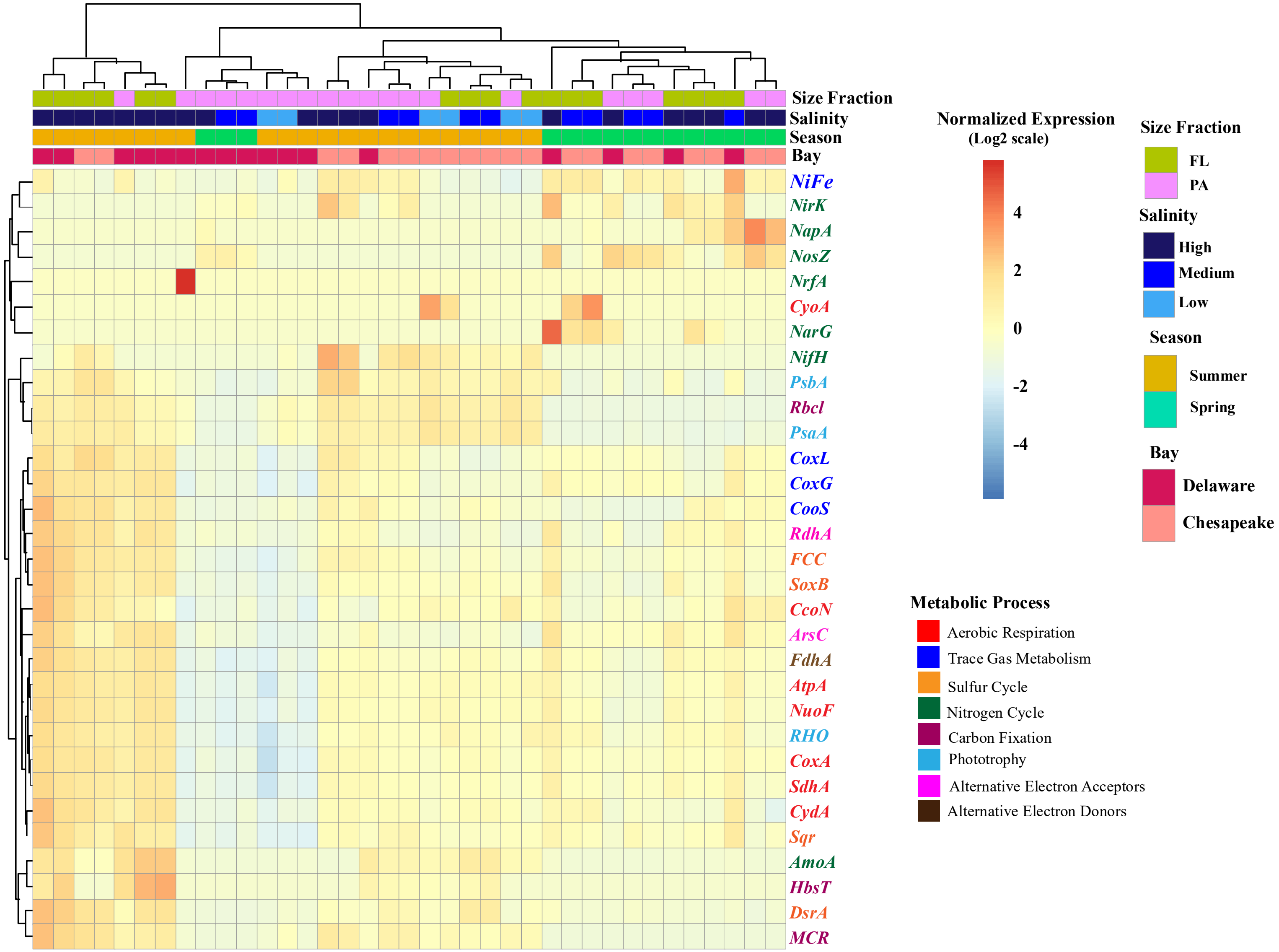

Supplementary Figure S4. Normalized expression of different metabolic markers. Heatmap displays log2 RNA-seq counts for selected metabolic marker genes. Rows represent genes, columns represent samples, and colors indicate relative expression (row-scaled). Sample annotations (e.g., salinity, season) are shown above the columns. Abbreviations: CP = Chesapeake Bay; DE = Delaware Bay; Sum = Summer; SPR=Spring # =salinity in PSU; G08 = >0.8 µm and L08 = <0.8 µm size fractions; # =RNA1 or RNA2.

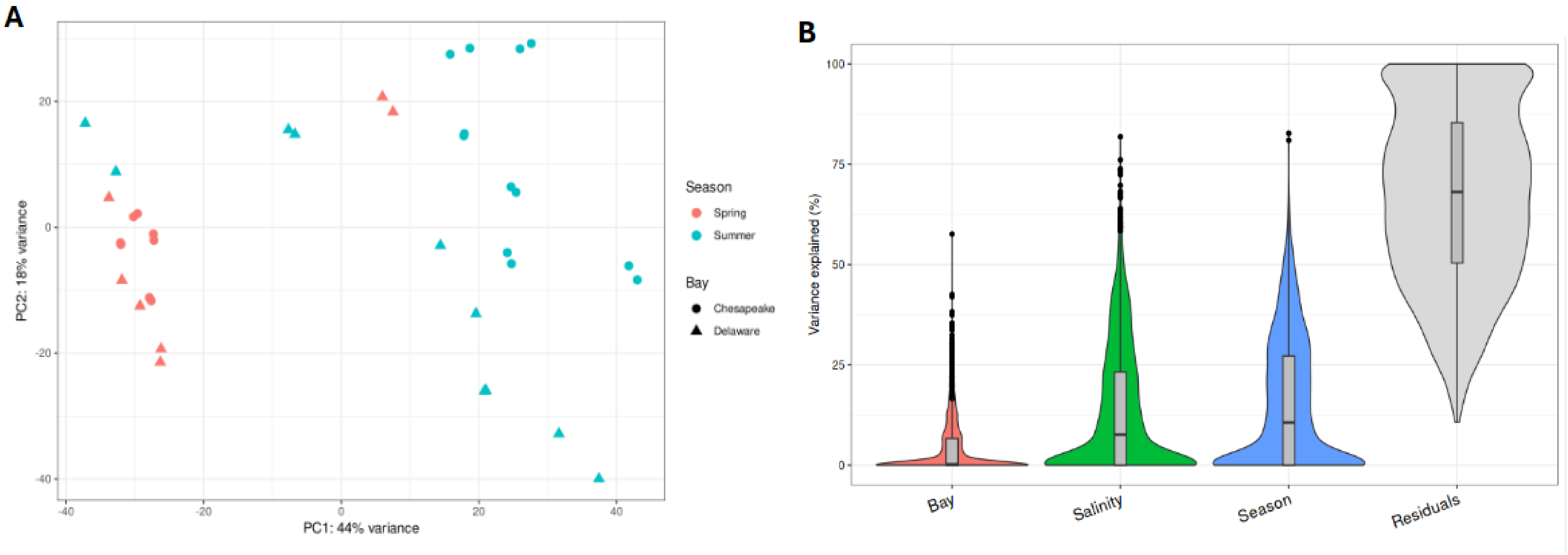

Supplementary Figure S5. PCA and variance partitioning of microbial communities. A. PCA of normalized expression data (vsd), with samples colored by season (red: Spring; blue: Summer) and shaped by bay (circle: Chesapeake; triangle: Delaware). PC1 and PC2 explain 44% and 18% of the variance, separating communities by season and bay. **B.** Variance partitioning showing the proportion of variance explained by Bay, Salinity, and Season. Violin plots illustrate the distribution across genes; residuals (unexplained variation) are shown for reference.

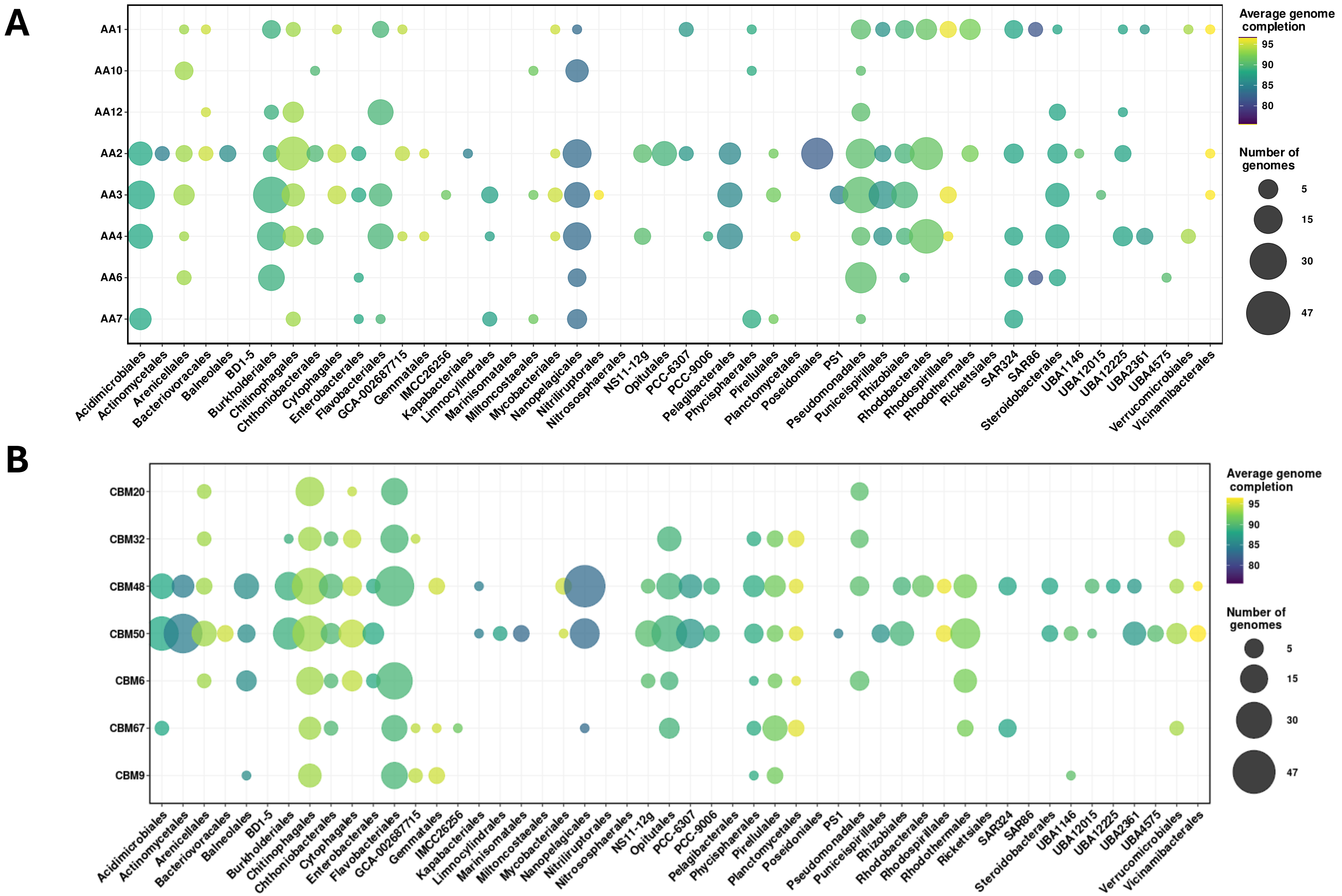

Supplementary Figure S6. Abundance of Auxiliary activity enzymes. A. and Carbohydrate-binding modules B. in the Chesapeake and Delaware MAGs. Combined MAG abundances at the order level are represented as bubble plots; Bubble size represents the normalized gene abundance, and hits were normalized based on thegenome completeness of MAGs from each order. CAZYmes were defined into different enzyme families, including GH (Glycoside hydrolase), GT (Glycosyl transferase), and CE (Carbohydrate esterase), which are shown on the y-axis.

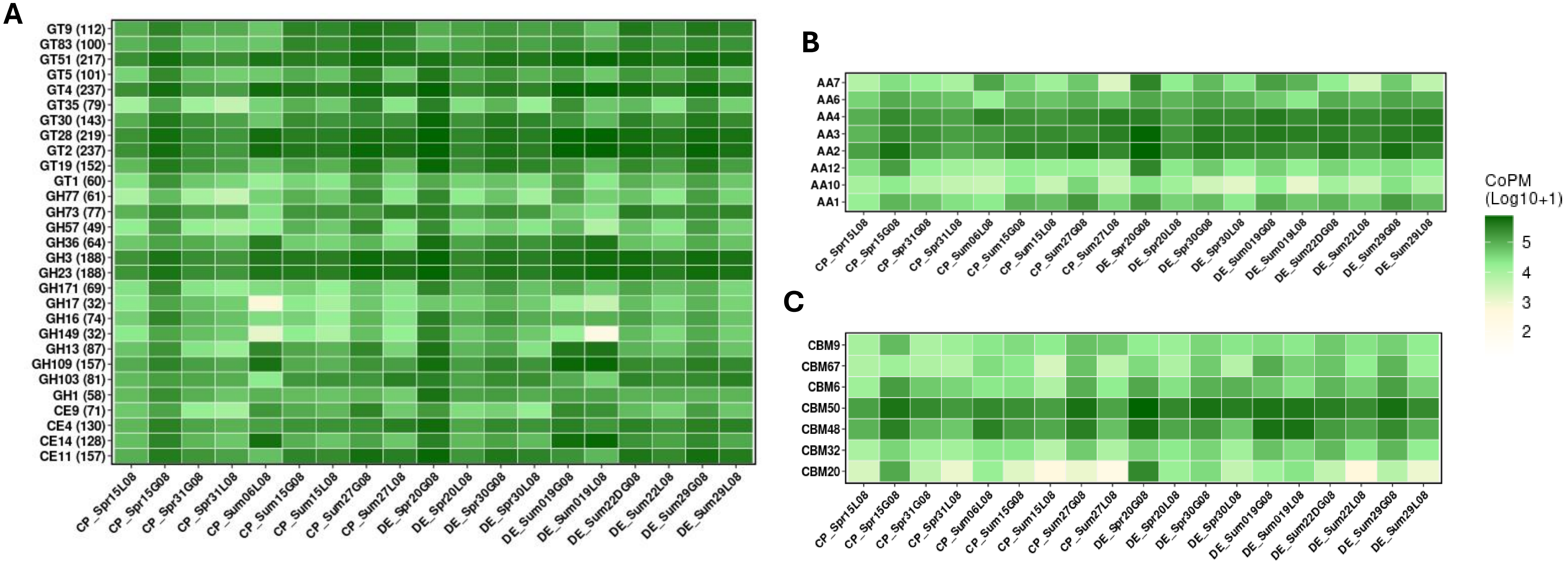

Supplementary Figure S7. CAZYmes, Auxiliary activity enzymes and Carbohydrate-binding modules in the Chesapeake and Delaware MAGs. A. Abundances of MAGs identified with each class of CAZYmes. The numbers in parentheses are the number of MAGs contributing to the corresponding CAZYmes**. B.** Abundances of Auxiliary activity enzymes (AAs). **C.** Abundances of Carbohydrate-binding modules (CBMs). CoPM represents the log-transformed abundance of MAGs that harbor the respective genes. CP = Chesapeake Bay, DE = Delaware Bay, Spr and Sum stand for the season, followed by salinity (06 PSU to 30 PSU) and size fraction (L08 less than 0.8 microns, G08 greater than 0.8 microns)

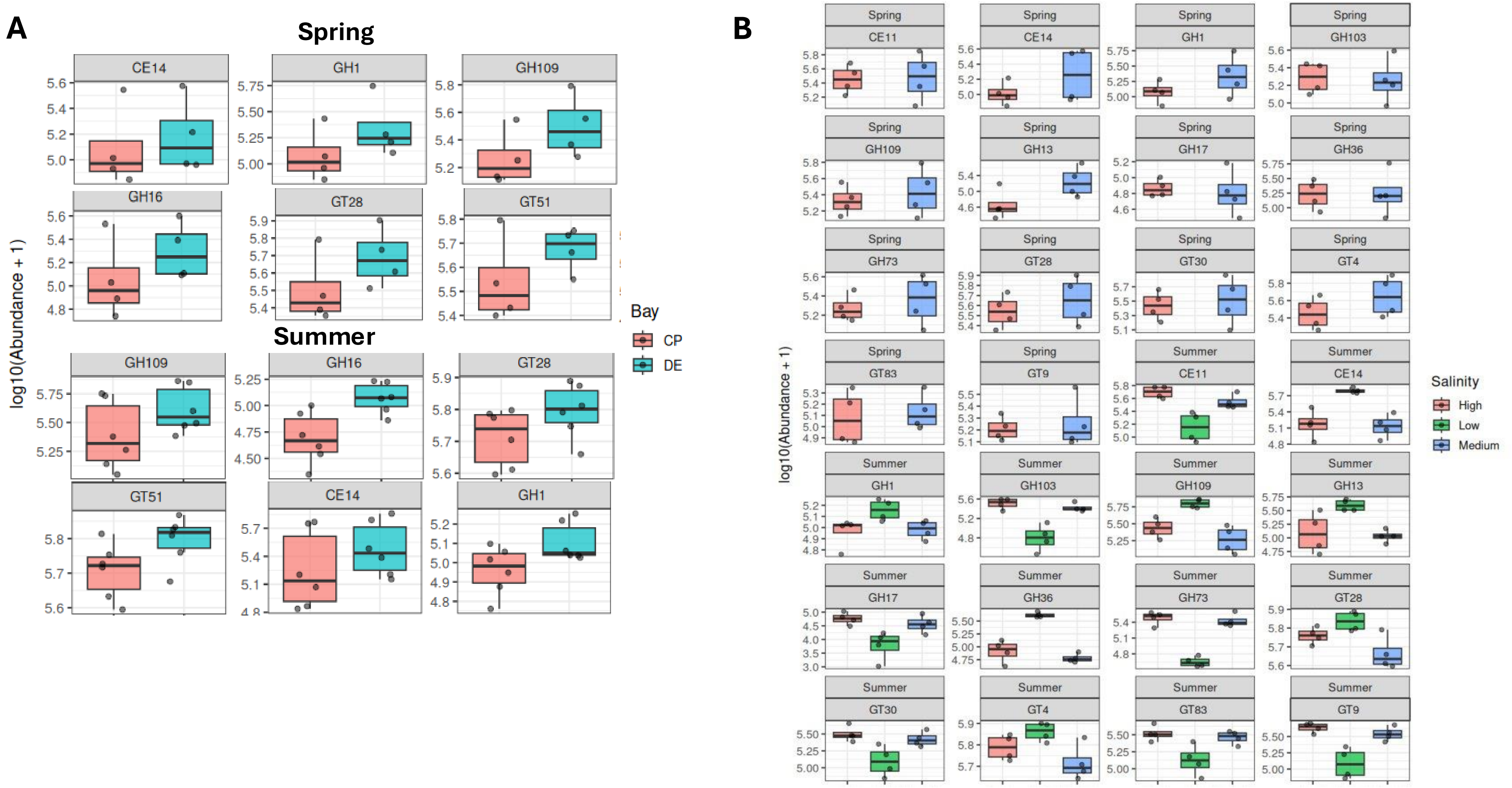

Supplementary Figure S8 Results of ARTS ANOVA showing the effects of Bay, Season, and salinity with their interaction on CAZyme abundance. A. CAZYmes with a statistically significant Bay effect, indicating differences in abundance between Chesapeake and Delaware Bays. **B.** CAZYmes with a statistically significant Season effect, highlighting changes in abundance between spring and summer under different salinities. Significance was determined based on adjusted *p*-values (FDR < 0.05).

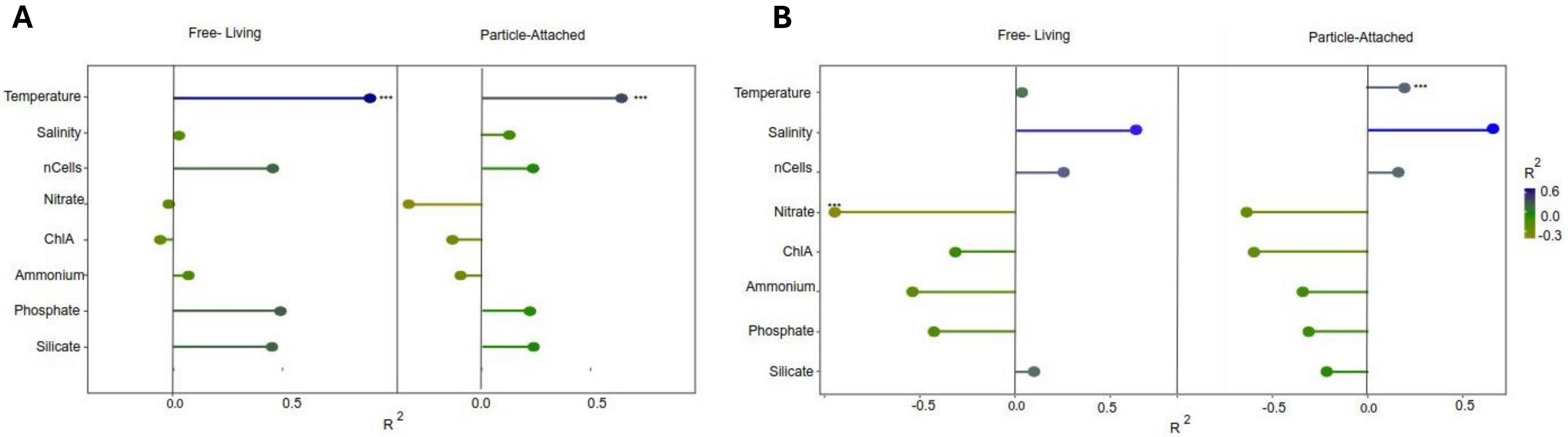

Figure S9: Drivers of functional redundancy in both bays. Correlation analysis of FRed with environmental factors across two different size fractions. **A.** Potential FRed. **B.** Expressed FRed. The R_2 value shows the relationship between the FRed and different environmental variables, ranging from 0 to 1, 0 indicating no variance and 1 indicating high variance. The different colors indicate the strength of the correlation, with darker colors corresponding to higher R-squared values. Statistically significant parameters ($p < 0.05$) are marked with asterisks on the figure

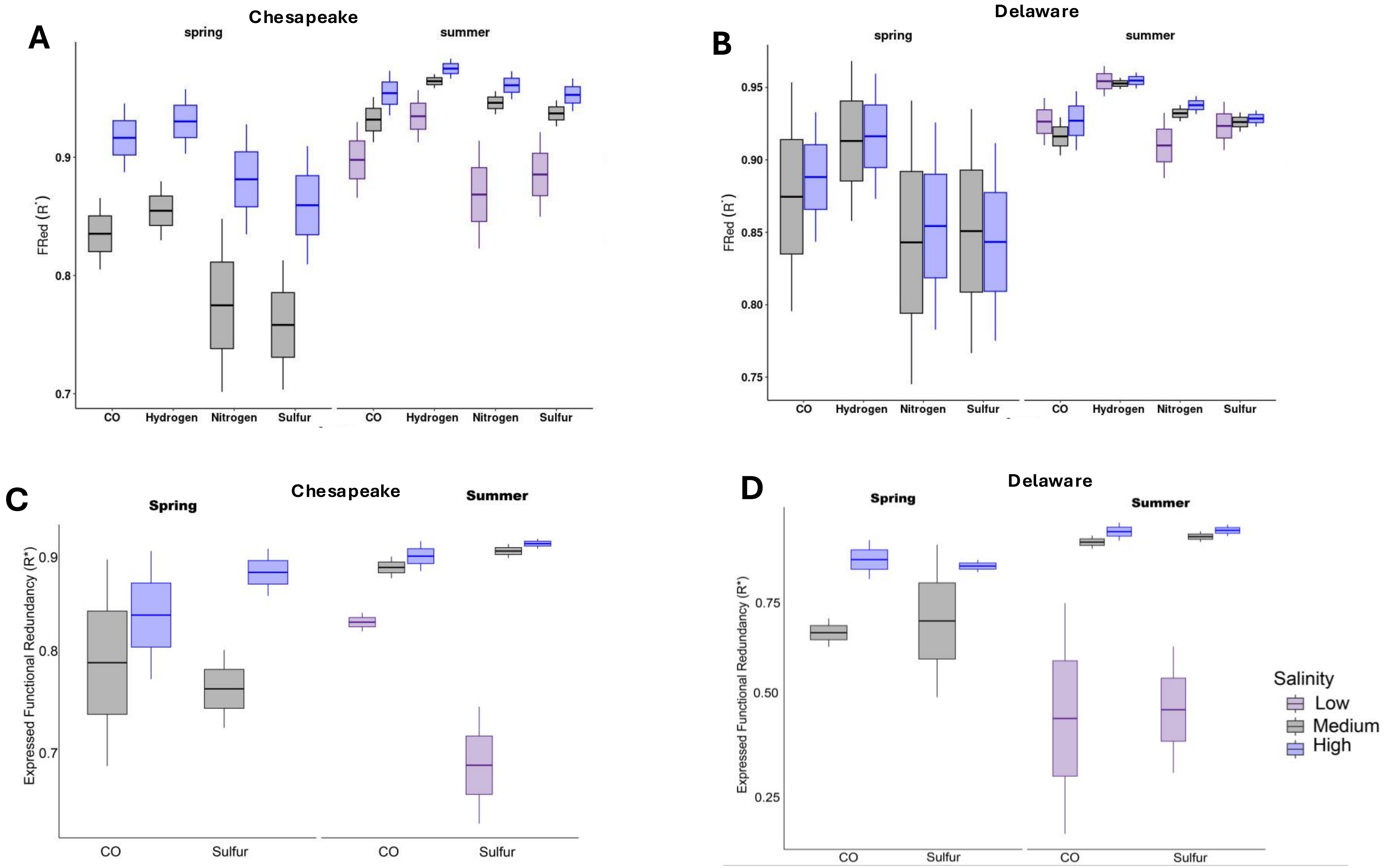

Supplementary Figure S10 Potential FRed and Expressed functional redundancy of metabolic modules. Potential and Expressed FRed in metabolic modules. Potential FRed in the Chesapeake Bay **A.** and Delaware Bay **B**. Expressed FRed in the Chesapeake Bay **C.** and Delaware Bay **D**. Different colors represent different salinities. The y-axis scale of both panels is not identical due to differences in the values.

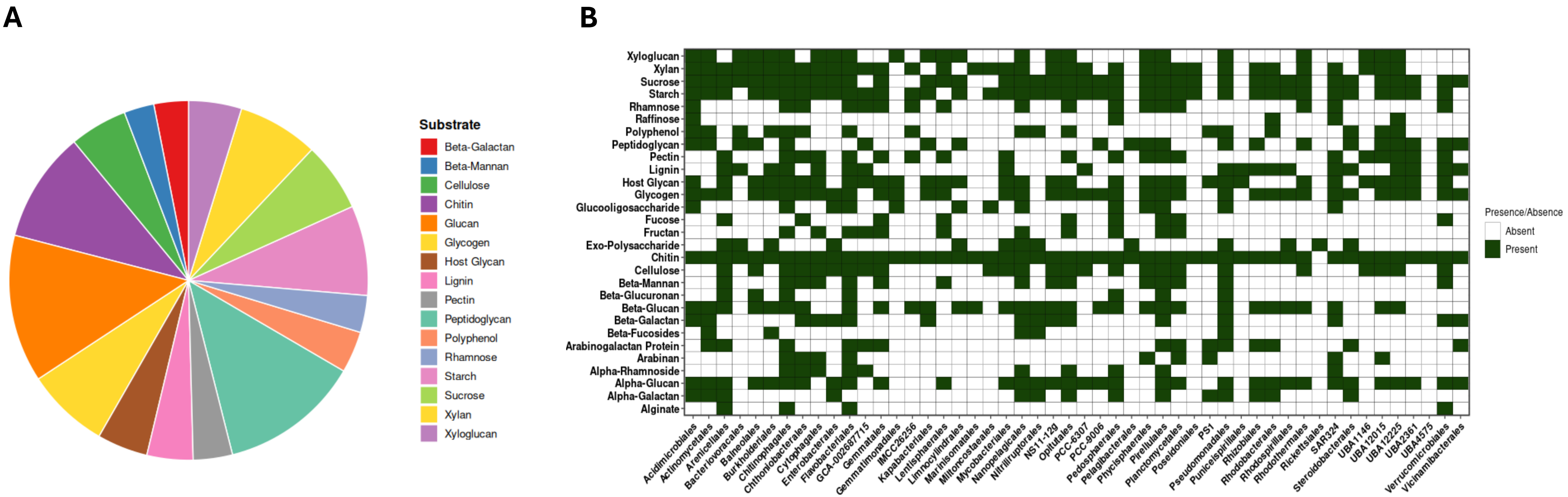

Supplementary Figure S11. CAZYme categories and substrates in the Chesapeake and Delaware MAGs. A. Number of MAGs possessing CAZYmes for the listed specific substrates. **B.** List of the most common substrates potentially utilized by MAGs, based on gene annotations, concatenated at the order level.

References

1. Ahmed MA, Lim SJ, Campbell BJ. Metagenomes, metatranscriptomes, and metagenome-assembled genomes from Chesapeake and Delaware Bay (USA) water samples. *Microbiology Resource Announcements*. 2021;10(21):10.1128/mra. 00262-21.

2. Maresca JA, Miller KJ, Keffer JL, Sabanayagam CR, Campbell BJ. Distribution and diversity of rhodopsin-producing microbes in the Chesapeake Bay. *Applied and environmental microbiology*. 2018;84(13):e00137-18.

3. Uritskiy GV, DiRuggiero J, Taylor J. MetaWRAP—a flexible pipeline for genome-resolved metagenomic data analysis. *Microbiome*. 2018;6:1-13.

4. Parks DH, Imelfort M, Skennerton CT, Hugenholtz P, Tyson GW. CheckM: assessing the quality of microbial genomes recovered from isolates, single cells, and metagenomes. *Genome research*. 2015;25(7):1043-1055.

5. Olm MR, Brown CT, Brooks B, Banfield JF. dRep: a tool for fast and accurate genomic comparisons that enables improved genome recovery from metagenomes through de-replication. *The ISME journal*. 2017;11(12):2864-2868.

6. Aroney ST, Newell RJ, Nissen JN, Camargo AP, Tyson GW, Woodcroft BJ. CoverM: read alignment statistics for metagenomics. *Bioinformatics*. 2025;41(4):btaf147.

7. Chaumeil P-A, Mussig AJ, Hugenholtz P, Parks DH. GTDB-Tk v2: memory friendly classification with the genome taxonomy database. *Bioinformatics*. 2022;38(23):5315-5316.

8. Langmead B, Salzberg SL. Fast gapped-read alignment with Bowtie 2. *Nature methods*. 2012;9(4):357-359.

9. Love MI, Huber W, Anders S. Moderated estimation of fold change and dispersion for RNA-seq data with DESeq2. *Genome biology*. 2014;15(12):550.

10. Ahmed MA, Campbell BJ. Genome-resolved adaptation strategies of Rhodobacterales to changing conditions in the Chesapeake and Delaware Bays. *Applied and Environmental Microbiology*. 2025:e02357-24.

12. Zhou Z, Tran PQ, Breister AM, Liu Y, Kieft K, Cowley ES, et al. METABOLIC: high-throughput profiling of microbial genomes for functional traits, metabolism, biogeochemistry, and community-scale functional networks. *Microbiome*. 2022;10(1):33.

13. et al. Integrating taxonomic signals from MAGs and contigs improves read annotation and taxonomic profiling of metagenomes. *Nature Communications*. 2024;15(1):3373.

14. Cram J, Hollins A, McCarty AJ, Martinez G, Cui M, Gomes M, et al. Microbial diversity and abundance vary along salinity, oxygen, and particle size gradients in the Chesapeake Bay. *Environmental Microbiology*. 2024;26(1):e16557.

15. Campbell BJ, Kirchman DL. Bacterial diversity, community structure and potential growth rates along an estuarine salinity gradient. *The ISME journal*. 2013;7(1):210-220.

16. Bouvier TC, del Giorgio PA. Compositional changes in free-living bacterial communities along a salinity gradient in two temperate estuaries. *Limnology and oceanography*. 2002;47(2):453-470.

17. Huang Y, He X, Lian Z, Yang Z, Jiang Q. Mapping the landscape of marine cultural heritage research from 2000 to 2023: A bibliometric analysis. *Marine Policy*. 2024;163:106086.

18. et al. Ecophysiology and genomics of the brackish water adapted SAR11 subclade IIIa. *The ISME journal*. 2023;17(4):620-629.
